# Structure and functional interactions of INO80 actin/Arp module

**DOI:** 10.1093/jmcb/mjy062

**Published:** 2018-11-02

**Authors:** Xuan Zhang, Xuejuan Wang, Zhihui Zhang, Gang Cai

**Affiliations:** 1 Hefei National Laboratory for Physical Sciences at Microscale and School of Life Sciences, University of Science & Technology of China, Hefei, China; 2 CAS Center for Excellence in Molecular Cell Science, Chinese Academy of Sciences, Hefei, China

**Keywords:** INO80, nuclear actin/Arp module, modular architecture, actin/Arp–Nuc207 assembly

## Abstract

The presence and functions of nuclear actin have been controversial due to the lack of molecular mechanisms. Nuclear actin and actin-related proteins (Arps) are subunits of several chromatin remodelers, including the evolutionarily conserved INO80 chromatin-remodeling complex. Here, we present an improved cryo-EM structure of the yeast INO80 complex and the first 3D reconstruction of the INO80 actin/Arp module. The modular and subunit architecture is defined using a combination of subunit deletion analysis and published crosslinking-mass spectrometry. The functional interactions of the INO80 actin/Arp module with a nucleosome is 3D EM reconstructed in two different binding states. Nucleosomes initially bind to the Arp8 subunit and the substantial conformational changes maximize nucleosome contacts of the actin/Arp module, which could promote the bound nucleosome to be engaged onto the INO80 ATPase domain. Our findings suggest that the conserved nuclear actin/Arp module acts a conformational switch of the INO80 for nucleosome binding.

## Introduction

The basic unit of chromatin organization is the nucleosome, which comprises 147 bp of DNA wrapped around a core of histone proteins (Kornberg, 1974; Luger et al., 1997). Chromatin structure critically regulates all nuclear processes that require access to DNA, including transcription, DNA replication, recombination, and repair. Chromatin modification is generally carried out by (i) covalent histone modifications by specific enzymes, such as acetylation, phosphorylation, methylation, ubiquitination, and so forth (Jenuwein and Allis, 2001; Peterson and Laniel, 2004) and (ii) ATP-dependent chromatin-remodeling complexes that specifically disrupt histone–DNA interactions by sliding, ejecting, or restructuring the nucleosomes (Smith and Peterson, 2005; Clapier and Cairns, 2009; Bartholomew, 2014).

ATP-dependent chromatin-remodeling complexes are characterized by the presence of an ATPase subunit belonging to the superfamily II helicase-related proteins (Singleton and Wigley, 2002; Flaus et al., 2006). An ATPase domain is comprised of two parts, the DExx/RecA1 and HELICc/RecA2 regions, which are separated by a linker. This superfamily can be further classified into at least four different families (SWI/SNF, ISWI, NURD/Mi-2/CHD, and INO80) based on the additional presence of unique domains within or adjacent to the ATPase domain (Flaus et al., 2006; Bartholomew, 2014). Interestingly, chromatin research in the past two decades has identified several large multi-subunit chromatin modifying or remodeling complexes containing nuclear actin and/or nuclear actin-related proteins (Arps), such as BAF, INO80, SWR1, and NuA4 (TIP60 in higher organisms) (Papoulas et al., 1998; Zhao et al., 1998; Galarneau et al., 2000; Shen et al., 2000; Mizuguchi et al., 2004). Excitingly, a recent study suggests a novel monomeric actin mechanism in INO80 complex in contrast to cytoplasmic actin (Kapoor et al., 2013). Given that the presence and function of nuclear actin have been controversial for decades due to the lack of defined molecular insights, the nuclear actin/Arp module provides a unique opportunity to understand the molecular mechanisms of nuclear actin (Harata et al., 2000). Since actin is a well-known conformational switch, one hypothesis is that nuclear actin/Arps function as conformational switches that control either the activity or the assembly of chromatin-remodeling machines (Boyer and Peterson, 2000).

INO80 chromatin-remodeling complex, which is evolutionarily conserved from yeast to man, contains actin and three Arps (Arp4, Arp5, and Arp8). Actin, Arp4, and Arp8 together with the helicase-SANT associated (HSA) domain of the Ino80 ATPase form a stable sub-module of INO80 (Shen et al., 2003; Szerlong et al., 2008), while Arp5 together with INO80 subunits Ies2 and Ies6 associate with the AAA^+^ ATPase subunits Rvb1 and Rvb2 (Jonsson et al., 2004; Chen et al., 2011; Tosi et al., 2013). Other INO80 subunits are Ino80 subunit (Ies) 1–6, TATA-binding-protein-associated factor 14 (Taf14) and high mobility group (HMG) domain-containing non-histone protein 10 (Nhp10) (Shen et al., 2003; Tosi et al., 2013). In total, *Saccharomyces cerevisiae* INO80 complex contains 15 different subunits and the molecular weight is over 1 MDa, which has been suggested to be organized into several different modules (Tosi et al., 2013). Ino80 acts as an assembly scaffold. The long insertion inside the conserved ATPase domain is responsible for the recruitment of the Rvb1/Rvb2 helicase (Jonsson et al., 2004), which is critical for assembly of the Arp5/Ies6 module (Chen et al., 2011). The Nhp10 module (Nhp10–Ies1–Ies3–Ies5) is mainly interacting with the N-terminal of Ino80 (Tosi et al., 2013). The actin/Arp module comprising the evolutionarily conserved subunits Act1, Arp4, Ies4, Taf14, and Arp8 associates with the HSA domain and N-terminal of the Ino80 (Kapoor et al., 2013; Tosi et al., 2013).

Both the INO80 and SWR1 complex were proposed to support similar functions and to share several subunits (Gerhold and Gasser, 2014); however, recent cryo-EM analyses by the Hopfner and Leschziner groups suggested the subunits stoichiometry, modular architecture and general topology of INO80 and SWR1 are substantially different (Nguyen et al., 2013; Tosi et al., 2013). The negative-stain study by the Walz and Peterson groups resolved the Rvb1/Rvb2 stoichiometry ambiguity (Watanabe et al., 2015). The human INO80 structure was recently determined at sub-nanometer resolution and illuminated the functional interaction of RUVBL1 and RUVBL2 with Ino80 and Ies2 (Aramayo et al., 2018). The structures of both human and yeast INO80 in complexes with a nucleosome offered very exciting insights into how INO80 the nucleosome binding of INO80 and how it catalyzes nucleosome sliding and histone editing (Ayala et al., 2018; Eustermann et al., 2018).

The INO80 actin/Arp module has been suggested to initiate the nucleosome binding of INO80 by associating with extranucleosomal DNA (Kapoor et al., 2013) and histones (Harata et al., 1999; Shen et al., 2003; Gerhold et al., 2012; Saravanan et al., 2012). As such, the actin/Arp module in INO80 provides a platform to reveal evolutionarily conserved molecular mechanisms for nuclear actin. However, until now, the detailed architecture of the actin/Arp module and nucleosome binding remains largely unknown. To determine the detailed architecture and how INO80 initiate nucleosome binding, we optimized the biochemical preparation and determined an improved cryo-EM reconstruction of the INO80 complex from *S. cerevisiae* and the first 3D reconstruction of the actin/Arp module in different conformational states. The modular and subunit architecture of the INO80 complex is further defined through the 3D reconstructions of several subunit deletion mutants. In addition, we 3D reconstructed the actin/Arp-Nucleosome assembly in different binding states. These analyses suggest the actin/Arp module in the INO80 complex serves as a conformational switch regulating nucleosome binding. Given that INO80 is one of the most evolutionary conserved chromatin-remodeling complexes, our findings on its actin/Arp module provide a novel platform to reveal the fundamental mechanisms of nuclear actin and Arps in regulating chromatin structure.

## Results

### Biochemical preparation of the INO80 complex

**Figure 1:**
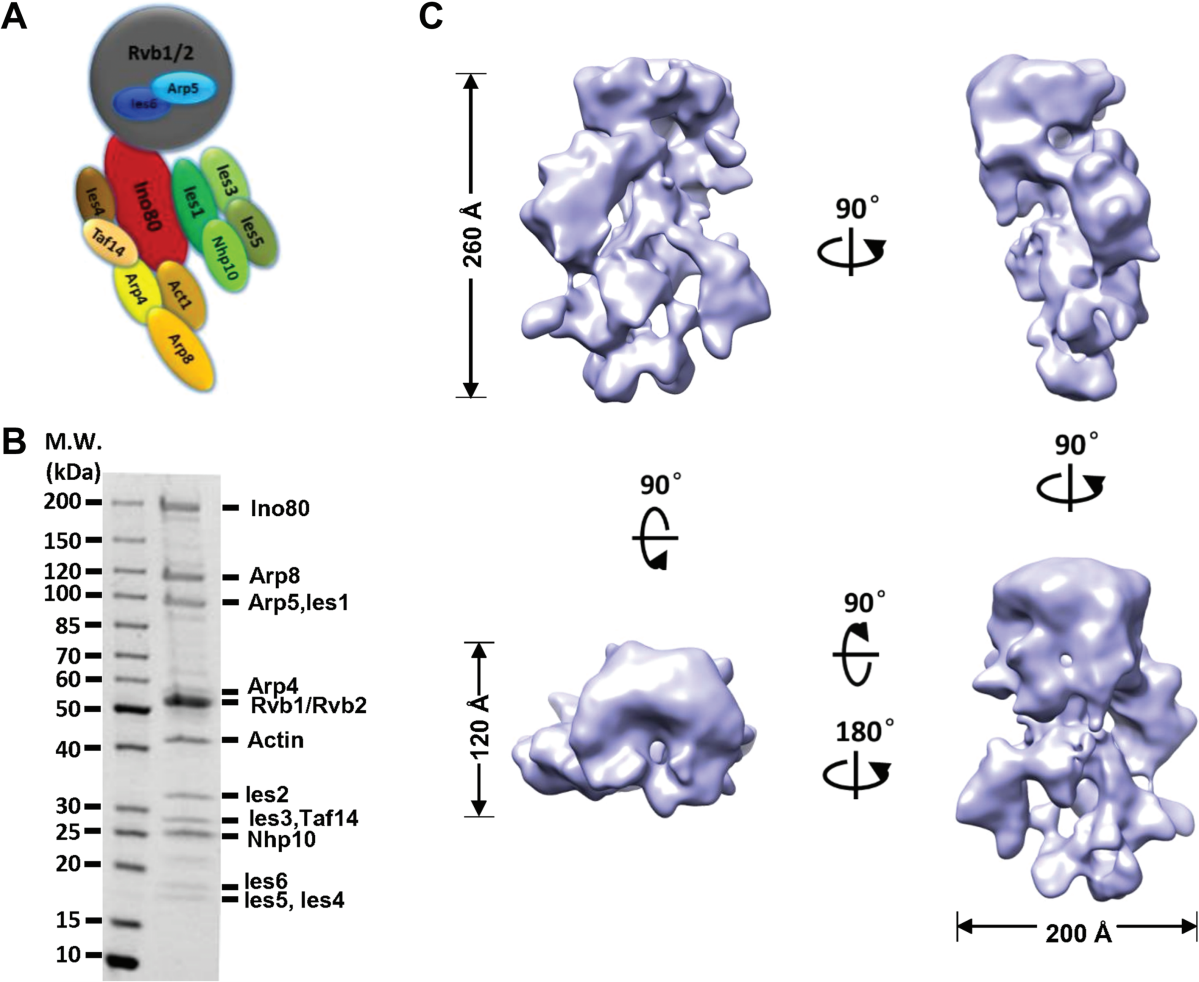
Structure of the yeast INO80 complex. (**A**) Schematic view of the subunit and modular organization of the INO80 complex from *S. cerevisiae*. (**B**) SDS-PAGE analysis of the endogenously purified INO80 complex. (**C**) Different views of the cryo-EM reconstruction of INO80 complex. Each successive view is rotated as indicated.

Due to the high composition complexity (Figure 1A) and flexibility of INO80 family chromatin-remodeling complexes, past structural analyses have been limited to low resolution structures that suffer from deformation artifacts induced by chemically crosslinking and by staining with heavy metals (Nguyen et al., 2013; Tosi et al., 2013; Watanabe et al., 2015; Lin et al., 2017). Previously, we established an efficient purification procedure to purify protein complexes endogenously involving ammonium sulfate precipitation to enrich the target-containing fraction (Cai et al., 2009). To further remove any minor contamination after the FLAG affinity chromatography, an ion exchange Mono Q column was employed. The improved procedure yielded the nearly stoichiometric 15-subunit yeast INO80 complex, which was uniform in composition based on SDS-PAGE analysis (Figure 1B). The particles observed by EM appeared well-preserved and were similar in size and overall shape (Supplementary Figure S1). To further improve homogeneity, we systematically optimized the mild crosslinking conditions for the GraFix step (Kastner et al., 2008) by EM and 2D class averaging (data not shown). As such, we obtained highly homogeneous INO80 complex suitable for cryo-EM analysis and avoided staining particles with heavy metals during specimen freezing (Supplementary Figure S3).

### 3D reconstructions of the INO80 in three conformational states

First, we performed negative-stain EM analysis to characterize the structural variability and obtain an initial model of INO80 (Supplementary Figure S2). INO80 complex is quite variable in its structure, which is exemplified by a flexible ‘tail’ domain and a globular ‘head’ domain. Notably, the INO80 tail domain appeared to be the most flexible, leading to a continuum of extended and compacted conformations. This observation is consistent with previous negative-stain EM analysis (Tosi et al., 2013; Watanabe et al., 2015). Three major conformations (named open, collapsed, and intermediate) of the INO80 differing in the positions of the tip of the ‘tail’ domain to the center of globular ‘head’ (open 240 Å; collapsed 170 Å; intermediate 165 Å) were identified through the reference-free alignment and classification of EM images. To ensure that the different averages indeed represent different conformations of the INO80 complex and not just different views, we obtained 3D structures of each conformation from images of tilted stained particles using the random conical tilt (RCT) method (Radermacher, 1988). The published yeast INO80 3D structures most closely resemble the open conformation (Tosi et al., 2013; Watanabe et al., 2015). These maps reveal head and tail features and confirm that the tail can adopt different conformations (Supplementary Figure S2). This result confirms and extends findings from previous EM studies in term of the overall shape of INO80.

### Improved Cryo-EM reconstruction

High-resolution images were recorded on a Titan Krios TEM equipped with a Falcon II camera (Supplementary Figure S3A). Classification of raw cryo-EM particles resulted in well-resolved 2D class averages, with the Rvb1-Rvb2 ring clearly discernable (Supplementary Figure S3B). After 3D classification of particles (Supplementary Figure S4), a subset of particles was subjected to high-resolution refinement, resulting in a 3D density map with the final overall resolution of 13.1 Å (Gold-standard FSC 0.143 criterion) (Supplementary Figure S5). The dimensions of INO80 are found to be 260 Å by 200 Å by 120 Å (Figure 1C). The close correspondence between projections of the INO80 cryo-EM structure and the 2D reference-free class averages corroborates the accuracy of the 3D cryo-EM reconstruction (Supplementary Figure S6). The 13.1 Å cryo volume is considerably more intricate and allows an unprecedented view of yeast INO80 structural organization. The structure of the head and tail regions, which were largely featureless in the stained reconstruction but now appear complex, displays several interconnected segments. Importantly, the structure revealed that the head domain could only contain Rvb1/Rvb2 hexameric ring, which is consistent with the recent observations (Watanabe et al., 2015; Aramayo et al., 2018; Ayala et al., 2018; Eustermann et al., 2018). Moreover, a protrusion spanning the globular ‘head’ is markedly more evident in the cryo-EM structure.

### EM analysis of INO80 Arp8Δ and Ies1Δ mutants

Previous EM analysis suggested that the INO80 actin/Arp module is localized at the tail domain (Tosi et al., 2013; Watanabe et al., 2015) and the Nhp10 module forms the body domain connecting the tail and the head domains (Tosi et al., 2013). Based on this model, the Nhp10 module should harbor multiple interactions with the actin/Arp and Rvb1/Rvb2 ring plus the Arp5 module. However, the interaction map of INO80 subunits revealed by XL-MS analysis clearly demonstrated that the Nhp10 module interacts only with N-terminal of Ino80 and Ies2 subunits and does not interact with any subunit of the actin/Arp and Arp5 modules (Tosi et al., 2013). These apparent contradictions suggest that the modular structure of the INO80 complex remains undefined. To illuminate the detailed modular architecture of the INO80, we 3D reconstructed the INO80 sub-complexes omitting the actin/Arp or Nph10 modules, to facilitate the identification of these INO80 modules.

**Figure 2:**
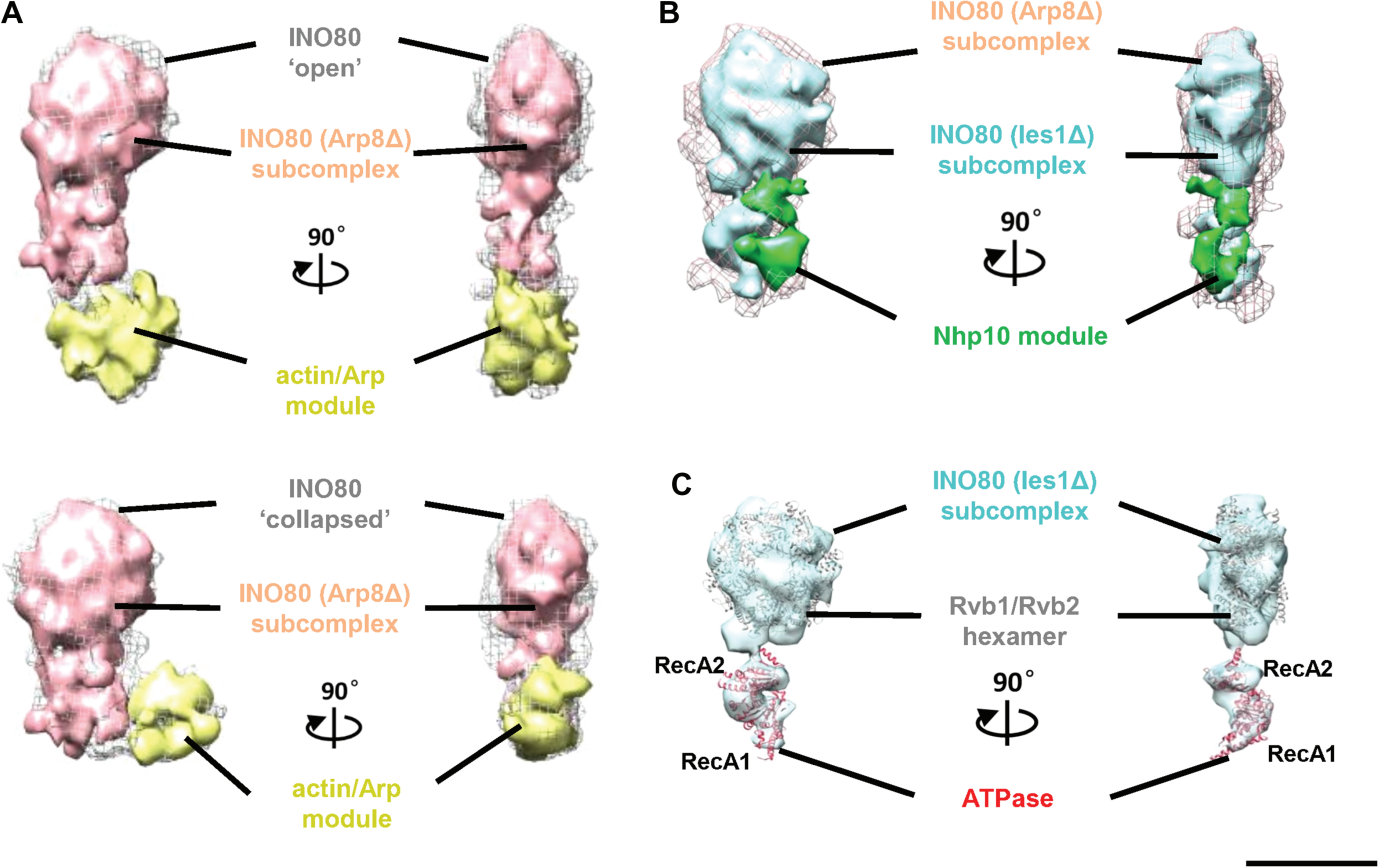
Identification of the actin/Arp and Nhp10 modules and the ATPase domain. (**A**) Comparison of the structure of the INO80 subcomplex (Arp8Δ, hot pick surface) with that of the INO80 complex in open and collapsed conformations (gray mesh), and difference mapping (yellow surface) identifies the actin/Arp module in both INO80 conformers. (**B**) Comparison of the structure of the INO80 (Ies1Δ) subcomplex (sky blue surface) with that of the INO80 (Arp8Δ) subcomplex (hot pink mesh), and difference mapping is calculated by subtracting the density of the Ies1Δ subcomplex from that of the Arp8Δ subcomplex, which highlights the boundary of the Nhp10 module (green surface). (**C**) Rigid-body fitting the crystal structural models of the human hexameric Rvb1/2 rings (PDBID:2XSZ) and the RecA domain of Mt Snf2 in the rest state (PDBID:5HZR) into the structure of the INO80 (Ies1Δ) subcomplex. The correlation coefficient value of rigid-body fitting Rvb1–Rvb2 (PDBID:2XSZ) and the ATPase domain of Mt Snf2 (PDBID:5HZR) is 0.93 and 0.98, respectively.

Firstly, we determined the structure of native INO80 missing the actin/Arp module from the Arp8Δ yeast strain (Supplementary Figure S7) and compared it with the INO80 holoenzyme. The structure matches that of the ‘head’ portion of the intact INO80 complex in both the open and collapsed conformations, and difference mappings identified the flexible ‘tail’ domain at the base as the actin/Arp module (Figure 2A). The identification of the actin/Arp module as the flexible tail is consistent with the previous EM analysis (Tosi et al., 2013; Watanabe et al., 2015; Aramayo et al., 2018). Furthermore, the detailed structural comparison of the Arp8Δ mutant with the INO80 holoenzyme confirms that the ‘head’ and ‘body’ part of the INO80 complex is largely stable. As such, the INO80 structural variability is predominantly due to changes in the positions of the highly mobile actin/Arp ‘tail’ at the bottom, which could be either far away from or abutted to the upper portion.

Further information about the boundary of the Nhp10 module was derived from a more detailed analysis of the structure of the Ies1Δ deletion mutant in which both the Nhp10 and actin/Arp modules dissociate (Supplementary Figure S8). Comparison of the 2D and 3D structures of the Arp8Δ mutant and those of the Ies1Δ indicates that the Nhp10 module matches the right-side portion of the ‘neck’ density in size and shape. Moreover, difference mapping identified the right-side density as the Nhp10 module (Figure 2B). The identification of the Nhp10 module residing on a peripheral position of the neck region is fully consistent with the Nph10 module harbors limited interaction with Ino80 and Ies2 revealed by XL-MS analysis (Tosi et al., 2013). Our mutational studies thus provide clear definition of the Nhp10 module in INO80.

Interestingly, the crystal structure of the catalytic core of *Myceliophthora thermophila* Snf2 in the resting state (PDBID:5HZR) (Xia et al., 2016) could be rigid-body fitted into the left ‘C’ shaped region of the ‘neck’, with the size and shape closely matched (Figure 2C). This finding identifies the location of the catalytic core of the Ino80 subunit and indicates that the ATPase domain is kept in the resting state before nucleosome binding. Our observation also suggests that the catalytic core of the Ino80 is poised for its incoming substrates and substantial conformational changes in the two ATPase core domains are required for activation. A similar model has been proposed for the Snf2 remodeler (Liu et al., 2017). Together, our detailed comparison of the structures of the Arp8Δ and Ies1Δ mutants, combined with XL-MS analysis, revealed unprecedented details in INO80 architecture, leading to identifications of the actin/Arp and Nhp10 modules and the location and conformation of the Ino80 ATPase domain.

### A new modular architecture of the INO80 complex

During our biochemical preparation of the INO80 complex, we also acquired several INO80 sub-complexes with the Rvb1/Rvb2 hexamers and dodecamers (Supplementary Figures S9 and S10). Surprisingly, such INO80 subassemblies and the separated Rvb1/Rvb2 rings were stably co-purified with the INO80 complex not only in the FLAG affinity but also the MonoQ fractions, we could only remove most of the co-purified Rvbs in the Grafix step. Towards better understanding, the INO80 complex assembly mechanism, we also 3D reconstructed such INO80 sub-complexes and the Rvb1/Rvb2 rings in the MonoQ fraction (Supplementary Figure S9). Detailed analysis of the 3D structures of INO80 and its subassemblies further substantiated that only a single Rvb1/Rvb2 hexameric ring could be assembled into the INO80 complex (Supplementary Figure S10). Unexpectedly, there are 4 out of the 18 classes of particles corresponding to the Rvb1/Rvb2 dodecamer and one class of particles corresponding to Rvb1/Rvb2 hexamer (Supplementary Figure S9). Such large proportion of isolated Rvb1/Rvb2 are co-purified with INO80 complex, suggesting that Rvb1/Rvb2 dodecamer and hexamer equilibrium could play a role in the INO80 assembly. The discrepancy of the Rvb1/Rvb2 stoichiometry in the previous EM studies of INO80 could be probably due to the substantial portion of Rvb1/Rvb2 dodecamer in such INO80 preparations (Tosi et al., 2013; Watanabe et al., 2015).

Among the structures of the INO80 subassemblies, there are several volumes containing one protrusion on the Rvb1/Rvb2 hexameric ring. We compared the 2D and 3D EM structures of such volumes with that of Rvb1/Rvb2 hexamer and identified the extra density matches the size and shape of the Arp5/Ies6 module (Supplementary Figure S10). The protrusion on Rvb1/Rvb2 hexamer being the Arp5/Ies6 module is fully consistent with the subunit interaction map revealed by XL-MS analysis (Tosi et al., 2013), which only interacts with Rvb2. Therefore, we have identified the actin/Arp module, Nhp10 module, and Arp5/Ies6 from the INO80 structure.

These observations improved modular understanding of the INO80 complex (Tosi et al., 2013), and advanced the definition of INO80 modular architecture based on recent negative-stain EM analyses of yeast INO80 (Watanabe et al., 2015). We confirmed that only a single Rvb1/Rvb2 hexamer could be assembled into the INO80 complex and identified the actin/Arp, Nhp10, and Arp5/Ies6 modules. We also pinpointed the Ino80 ATPase domain. The localization of the ATPase domain substantiates that Ino80 subunit is the scaffold to assemble the whole INO80 complex, with the N-terminal binds to Nhp10 module, the HSA domain and its proceeding region interacts with actin/Arp module, and the long insertion in the RecA2 domain associates with Rvb1/Rvb2 hexameric ring, where the Arp5/Ies6 module binds (Supplementary Figure S10).

### Comparisons of the structure and subunit organization of the yeast and human INO80 complexes

**Figure 3:**
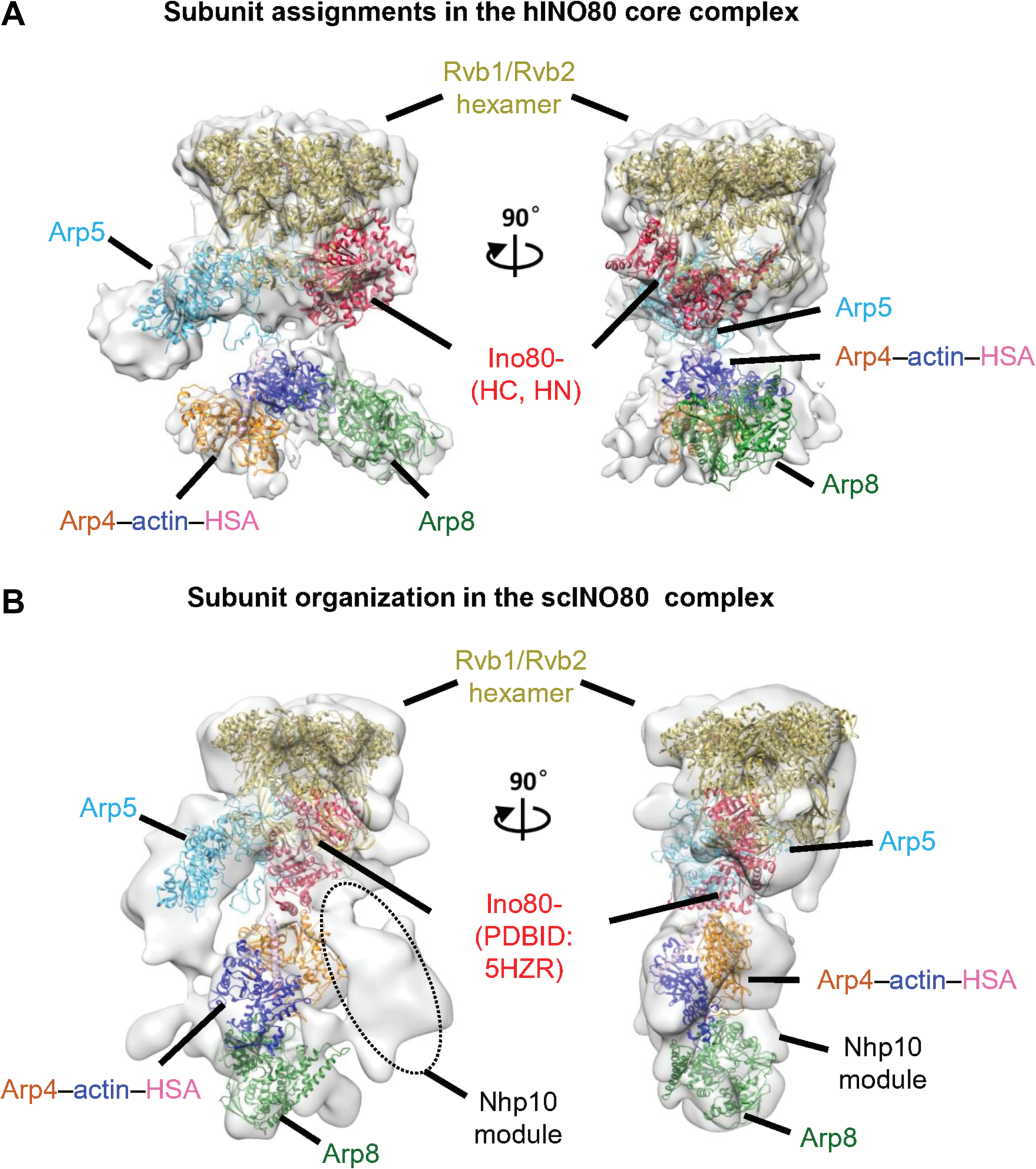
Modular organization of the INO80 complex. (**A**) The published cryo-EM structure and subunit architecture of the human INO80 complex (Aramayo et al., 2018). The correlation coefficient value of rigid-body fitting ATPase domain (PDBID:5HZR), Rvb1–Rvb2 (PDBID:5OAF), Arp5 homology model, Arp8 (PDBID:4AM6), and actin–Arp4–HSA (PDBID:5I9E) into human INO80 structure is 0.74, 0.91, 0.74, 0.61, and 0.74, respectively. (**B**) Our cryo-EM reconstruction and subunit organization of the yeast INO80 complex. The correlation coefficient value of rigid-body fitting ATPase domain (PDBID:5HZR), Rvb1–Rvb2 (PDBID: 5OAF), Arp5 homology model, Arp8 (PDBID:4AM6), and actin–Arp4–HSA (PDBID:5I9E) into yeast INO80 structure is 0.84, 0.89, 0.87, 0.90, and 0.90, respectively. The black dashed oval highlights the position of the Nhp10 module.

Fitting the available structures of the INO80 subunits into our yeast cryo-EM reconstructions, constrained by the combination of subunit deletion analysis and published CX-MS information (Tosi et al., 2013), allows us to derive a close examination of the INO80 assembly (Figure 3B). The structural model provides a platform to integrate the structural and functional information into a coherent mechanism for INO80 modularity and its chromatin-remodeling mechanism. We then compare the structure and subunit organization of our yeast INO80 with that of published human counterpart (Aramayo et al., 2018) and find the organization of the Rvb1/Rvb2 ring, INO80 ATPase and Arp5 is largely similar (Figure 3). Besides the Nhp10 module missing in the human INO80 preparation, the localizations of Arp4–actin–INO80 HSA and Arp8 show large variation in the yeast and human INO80 complexes (Supplementary Figure S11). Taking in account the intrinsic structural flexibility of the actin/Arp module and that the Nhp10 module is missing in human INO80, the yeast and human INO80 structures possibly recapitulate different conformational states of the actin/Arp module (Figure 3).

### 3D reconstructions of the INO80 actin/Arp module

**Figure 4:**
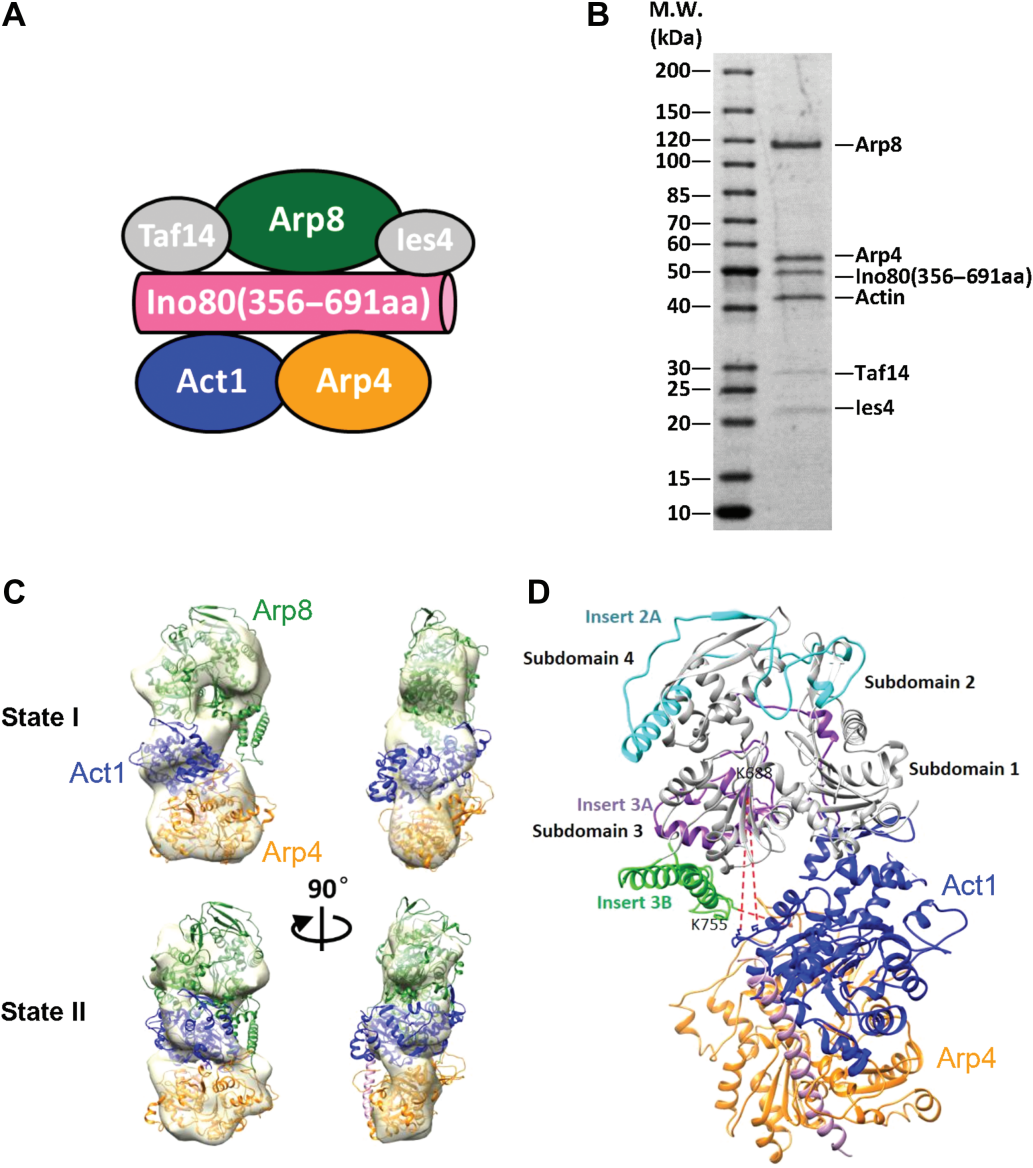
3D reconstruction of the INO80 actin/Arp module. (**A**) Schematic view of the subunit organization of the INO80 actin/Arp module. (**B**) SDS-PAGE of the endogenously prepared actin/Arp module. (**C**) Different views of two 3D reconstructions of the actin/Arp module with the crystal structural models of the Arp8 and actin–Arp4–HSA rigid-body fitted. The correlation coefficient value of fitting Arp8 (PDBID:4AM6) and actin–Arp4–HSA (PDBID:5I9E) into state I is 0.88 and 0.91 and into state II is 0.86 and 0.92, respectively. This location is in agreement with crosslinks of Arp8 to actin–Arp4–HSA (Tosi et al., 2013). (**D**) The pseudo-atomic model of the actin/Arp module constrained by the EM map and the subunit interaction map derived from the XL-MS study (Tosi et al., 2013). The two insertions of Inserts 3A and 3B in the Arp8 make critical contact with actin (Arp8 K688 with actin K326 and K328) and Arp4 (Arp8 K775 with Arp4 K218).

The INO80 actin/Arp module provides a defined system to reveal the elusive mechanisms of nuclear actin. Previous studies suggest that the actin/Arp module is functionally important for INO80 chromatin remodeling and directly interacts with DNA and histones (Shen et al., 2003; Kapoor et al., 2013; Tosi et al., 2013). However, the structural details of this key module remain unclear. Interestingly, the INO80 N-terminal subcomplex (N.com) could be endogenously purified from yeast cells, consisting of Ino80 (356–691 aa, named as N-FLAG), actin, Arp4, Arp8, and Taf14 (Kapoor et al., 2013). To better define the evolutionarily conserved actin/Arp module, and study its interaction with nucleosome substrates, we acquired the endogenous N.com and the SDS-PAGE analysis (Figure 4A and B) confirmed the presence of N-FLAG, actin, Arp4, Arp8, and Taf14. In addition, there is also stoichiometric Ies4 subunit co-purified, adding another component for the actin/Arp module (Tosi et al., 2013).

Given that actin is a well-known conformational switch, the structure of the N.com is indeed highly heterogeneous. We determined 3D structures of N.com in several conformations (Supplementary Figure S12). The available crystal structures of the Arp8 (PDBID:4AM6) (Saravanan et al., 2012) and actin–Arp4–HSA (PDBID:5I9E) (Cao et al., 2016) could be rigid-body fitted into these 3D volumes (Figure 4C). The fitting was in agreement with several crosslinks between Arp8 and actin/Arp4 (Arp8 K688–Actin 326; Arp8 K688–Actin 328, and Arp8 K775–Arp4 K218) (Figure 4D) (Tosi et al., 2013). After fitting the crystal structure of the Arp8 and actin–Arp4–HSA into the 3D map of the N.com, we gained the first structural glimpse of the assembly of the INO80 actin/Arp module. Arp8 associates with Ino80 through the sequence proceeding the HSA domain and its subdomain 1 constitutes the major contact region with actin. The two insertions within the actin-like domain of Arp8 (Saravanan et al., 2012) play a critical role in the actin/Arp module assembly, with Insert 3A to bind Arp4 and with Insert 3B to interact with actin (Figure 4D). The actin–Arp4–HSA are relatively stable, whereas the Arp8 could undergo a range of motion due to the intrinsic disordered linker (381–448 aa) of the Ino80 subunit, which binds to the N-terminal of Arp8 subunit. Our structural analysis therefore provides unprecedented details for the INO80 actin/Arp module and reveals a key role for Arp8 in organizing this module.

### Actin/Arp module is a switch for nucleosomes binding of INO80 complex

A major challenge in understanding mechanisms of chromatin remodeling has been the lack of structural insights into how a chromatin-remodeling complex engages nucleosomes. Such key advance requires the determination of structures of nucleosome-bound INO80 or different INO80 remodeling intermediate states. Therefore, we aimed to obtain initial structures of these intermediates. Departing from the Hopfner study that used the nucleosome core particle (Tosi et al., 2013), we used mononucleosomes with linker DNA (Nuc207, containing 30 bp linker DNA on each side of the nucleosome), which is known to bind INO80 with higher affinity (Kapoor et al., 2013). We found that actin/Arp module itself forms a stable complex with Nuc207 with a 1:1 stoichiometry in the absence of ATP, and we visualized the assembled actin/Arp–Nuc207 assembly by EM.

**Figure 5:**
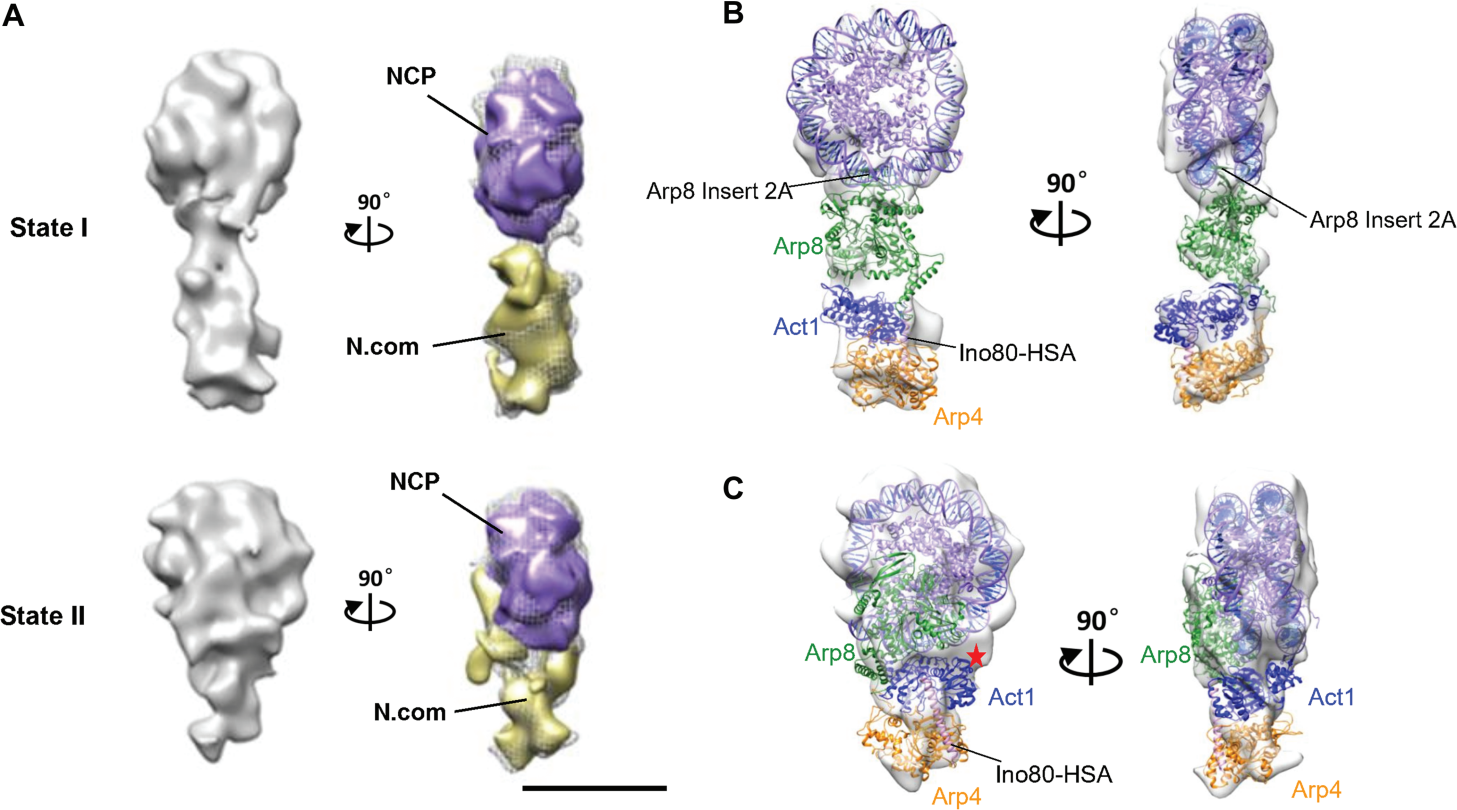
3D reconstruction of the actin/Arp–Nuc207 assembly. (**A**) 3D EM reconstructions of the actin/Arp–Nuc207 assembly in two different binding states. The actin/Arp densities (yellow) could be identified by subtracting the nucleosome densities (purple) in the right panel. Each successive view is rotated as indicated. (**B**) Different views of the 3D reconstruction of actin/Arp–Nuc207 assembly in state I, which is rigid-body fitted by the structural models of the nucleosome and actin/Arp module. The correlation coefficient value of fitting nucleosome (PDBID:2PYO), Arp8 (PDBID:4AM6), and actin–Arp4–HSA (PDBID:5I9E) into state I assembly is 0.91, 0.84, and 0.89, respectively. (**C**) Different views of the 3D reconstruction of actin/Arp–Nuc207 assembly in state II, which is rigid-body fitted by the structural models of the nucleosome and actin/Arp module. The correlation coefficient value of fitting nucleosome (PDBID:2PYO), Arp8 (PDBID:4AM6), and actin–Arp4–HSA (PDBID:5I9E) into state II assembly is 0.93, 0.79, and 0.80, respectively. The location is in agreement with crosslinks of Arp8 to actin–Arp4–HSA with the nucleosome (Tosi et al., 2013). The Arp8 Insert 2A is identified and the actin A58 residue is labeled with a red star.

We observed two different nucleosome binding states for the INO80 actin/Arp module and the density of the nucleosomal substrate is readily identified (Figure 5A and Supplementary Figure S13). In the first state, the Nuc207 binds to the peripheral of the actin/Arp module with the Insert 2A within the actin-like domain of Arp8 contacting the two gyres of nucleosomal DNA (Figure 5B). In another state, the Arp8 undergoes substantial conformational changes and expands on the exposed histone face of the nucleosome (Figure 5C). This state is consistent with the previous biochemical and structural observations of Arp8 interaction with nucleosome DNA and histones, especially the Insert 3A of Arp8 was shown to interact with H3/H4 tetramers (Gerhold et al., 2012; Saravanan et al., 2012). In accompany of the Arp8 conformational changes, actin and Arp4 also move towards the Nuc207 to directly interact with both the histone and DNA, which is in agreement of the XL-MS analysis of INO80 nucleosome complex (Tosi et al., 2013). Interestingly, the actin subdomain 2 directly binds to the nucleosome DNA with the A58 residue in close proximity to the DNA binding interface (Figure 5C). This observation is consistent with the finding that actin subdomain 2 is crucial for INO80 complex interaction with chromatin (Kapoor et al., 2013) and suggests that *act1-2* (A58T) mutant reducing DNA and nucleosome binding is probably due to the abrogate of the DNA binding interface.

## Discussion

The actin/Arp module has been implicated in nucleosome-binding of INO80 complex (Shen et al., 2003; Tosi et al., 2013; Watanabe et al., 2015), we found that the actin/Arp module stably binds to chromatin independently of the ATPase subunit with a 1:1 stoichiometry. These observations consistently suggest that the actin/Arp initiates the nucleosome binding for the INO80 complex. The 3D reconstructions of actin/Arp–Nuc207 in two different states represent the first structural glimpse of the cooperative nucleosome binding of the INO80 complex. The two binding states suggest that nucleosomes probably initially bind to the Arp8 subunit, which then undergoes the most substantial conformational changes and induces the synergistic conformational changes in the INO80 HSA domain, actin, and Arp4 to promote contacts of the nucleosome with the entire actin/Arp module. Therefore, the actin/Arp module, especially the Arp8 subunit constitutes a critical conformational switch to regulate nucleosomes binding and allow INO80 complex to bind cooperatively to nucleosomes. Given that Arp8 is unique to the INO80 class of remodeling complexes, it is likely that Arp8-coordinated actin/Arp dynamics represents a fundamental and conserved mechanism for nuclear actin. Moreover, although actin itself does not bind to DNA, our findings suggest that in concert with Arp4/Arp8 and other subunits in the module, nuclear actin is capable of directly interacting with chromatin.

The INO80 chromatin-remodeling complex is a critical regulator of transcriptional regulation, DNA replication, and DNA repair. Given the high degree of conservation from yeast to human (Chen et al., 2011), an integrated structural biology approach for INO80 study holds the promise to unravel the fundamental mechanisms of chromatin remodeling, as well as revealing the unique mechanisms of nuclear actin. We have now redefined the modular architecture of the yeast INO80 and its actin/Arp module by EM and integrated the structural and functional information into a coherent model for INO80 architecture and nucleosome-binding mechanism. This study thus constitutes a critical initial step towards establishing an integrated structural biology approach and providing a structural framework for understanding fundamental mechanisms of chromatin remodeling, as well as solving the mystery of nuclear actin.

## Materials and methods

### Purification of the INO80 complex and actin/Arp module

*S. cerevisiae* strains Ino80-FLAG *(MATa Ino80-2*×*FLAG his3Δ200 leu2Δ0 met15Δ0 trp1Δ63 ura3Δ0)* (Shen et al., 2000) and the INO80 (N.com)-FLAG *(MATa ino80Δ::TRP1 his3Δ200 leu2Δ0 met15Δ0 trp1Δ63 ura3Δ0 [pN(INO80)-2 XFLAG])* (Kapoor et al., 2013) were grown in YPD medium. About 100 g cells were harvested, washed and re-suspended in extraction buffer (250 mM Tris-HCl, pH 7.6, 100 mM KOAc, 300 mM ammonium sulfate (AS), 2 mM EDTA, 5 mM β-ME, 35% (*v*/*v*) glycerol and protease inhibitors) and a whole-cell extraction was prepared as previously described (Cai et al., 2009). This whole-cell extract was selectively precipitated in 30%–55% AS and re-suspended using FLAG Binding buffer (25 mM Tris-HCl, pH 7.6, 200 mM KOAc, 5 mM EDTA, 10% (*v*/*v*) glycerol, and protease inhibitors). After the suspension was clarified by centrifugation, the supernatant was incubated for 2 h at 4°C with 1 ml of 50% slurry of FLAG resin beads (GE Healthcare). After incubation, the beads were washed with 50 ml of wash I buffer (50 mM Tris-HCl, pH 7.6, 500 mM AS, 1 mM EDTA, 10 μM ZnCl_2_, 10% (*v*/*v*) glycerol, and protease inhibitors), followed by a second wash with 50 ml of wash II buffer (50 mM Tris-HCl, pH 7.6, 50 mM AS, 1 mM EDTA, 10 μM ZnCl_2_, 10% (*v*/*v*) glycerol, and protease inhibitors). After equilibration of the column with FLAG-elution buffer (50 mM HEPES, pH 7.6, 100 mM AS, 1 mM EDTA, 10 μM ZnCl_2_, 10% (*v*/*v*) glycerol), 150 ng/μl 3×FLAG Peptide was added to the resin beads and incubated 1 h at 4°C. The INO80 or actin/Arp module fraction was eluted with FLAG-elution buffer, respectively. The elution fractions were then further purified to near homogeneity by Mono Q ion exchange chromatography column (GE Healthcare) with Q 100 buffer (100 mM AS, 50 mM HEPES, 1 mM EDTA, 10 μM ZnCl_2_, 0.01% NP-40, 5 mM β-ME, 10% (*v*/*v*) glycerol, and protease inhibitors) and were resolved over a 100–1000 mM AS gradient. The INO80 or actin/Arp module peak fractions were flash-frozen in liquid nitrogen, analyzed by SDS-PAGE and EM examination.

### Purification of the INO80 subassemblies from the Arp8Δ and Ies1Δ mutants

The C-terminal of the Ino80 was affinity tagged with modified TAP tag (Cai et al., 2009) in the Arp8Δ and Ies1Δ mutant from the Homozygous Diploid Complete Set (Cat. no. 95401.H1R3, Invitrogen) and the purification method is the same with the WT INO80 complex.

### GraFix preparation of the INO80 complex

To further improve the homogeneity, we systematically optimized the mild crosslinking conditions for the GraFix step (Kastner et al., 2008) by EM and 2D class averaging. To form the gradient, we prepared a glycerol-glutaraldehyde gradient in a 4 ml ultracentrifuge tube. Briefly, 2 ml low gradient buffer (20 mM HEPES, pH 8.0, 100 mM KOAc, 5 mM MgCl_2_, 1 mM EDTA, 0.1% trehalose, 0.01% NP-40, 10% (*v*/*v*) glycerol, 2 mM DTT) was loaded into ultracentrifuge tube, and 2 ml high gradient buffer with 40% (*v*/*v*) glycerol and 0.02% glutaraldehyde containing the same buffer was carefully injected into the bottom of the tube using a blunt-end needle. The gradient was formed using a Gradient Master instrument and precooled at 4°C for 1 h. After adding 50 pmol of INO80 to the top of the gradient, the sample was ultracentrifuged in a SW 60 Ti rotor (Beckman) at 45000 rpm for 18 h at 4°C. Fractions (100 μl) were collected and the crosslinking was quenched with 5 M glycine, which were checked by negative-stain EM. The best fraction containing the most homogeneous INO80 complex was selected for cryo-EM analysis.

### EM sample preparation and RCT reconstruction

The GraFix-treated sample of INO80 complex was diluted five times and the Mono Q peak fraction of actin/Arp module was diluted four times with the EM buffer (20 mM HEPES, pH 8.0, 40 mM KOAc, 5 mM MgCl_2_, 0.1% trehalose, 2 mM dithiothreitol (DTT), and 0.01% NP-40). About 3 μl of aliquots were applied to a freshly glow-discharged carbon-coated 400-mesh Cu EM specimen grid, and then preserved by staining with 0.75% (*w*/*w*) uranyl formate solution. Images were recorded at a magnification of 62000× on a 4096 × 4096 charge-coupled device (CCD) detector (FEI Eagle) with a Tecnai F20 electron microscope (FEI) operating at an acceleration voltage of 200 kV, by using low-dose procedures with a defocus range of ~1.0–1.2 μm. Two-fold pixel binning of the original CCD images resulted in a final pixel size of 3.54 Å per pixel.

The 3D reconstructions under negative stain were calculated by using the RCT method (Radermacher, 1988). Tilted (−55°) and untilted (0°) image pairs were obtained under low-dose conditions, and particles were selected using the TiltPicker (Voss et al., 2009) and were montaged for interactive screening, yielding ~5800 tilt-pairs images of the INO80 and ~31000 tilt-pairs images of the actin/Arp module. We run iterative alternating rounds of supervised multi-reference alignment and classification as well as reference-free alignment to improve the homogeneity of the image classes. All the 3D reconstructions were calculated with SPIDER (Frank et al., 1996) and SPARX (Hohn et al., 2007).

### Sample vitrification and cryo-EM data collection

The INO80 fraction was diluted to a final concentration of ~30 μg/ml (20 mM HEPES, pH 8.0, 40 mM KOAc, 5 mM MgCl_2_, 0.1% trehalose, 2 mM DTT, and 0.01% NP-40) and 3 μl aliquots were applied to freshly glow-discharged Quantifoil R2/1 grids coated with a second layer of thin carbon film. The grids were blotted for 3–4 sec at 4°C in 100% humility, and then plunged into liquid ethane using an FEI Vitrobot (FEI Company). Frozen grids were stored in liquid nitrogen. The grids were first loaded into a Gatan 626 cryo-holder and transferred to an FEI Tecnai TF20 electron microscope to check the quality of the sample vitrification. Then, the grids were transferred to Titan Krios equipped with a field emission source and were operated at 300 kV. Images were recorded on a Falcon II direct electron detector at a nominal magnification of 59000 with a defocus range of 3–5 μm, resulting in a calibrated sampling of 1.42 Å per pixel. The total accumulated dose rate was set to be 35 e^2^ per Å^2^ on the specimen, and the exposure time was 1.5 sec. Each image was fractionated into 23 frames.

### Image processing

All micrographs were corrected for beam-induced drift using motion correction method (Li et al., 2013). CTF parameters and defocus values for each micrograph were determined by using CTFFIND3 (Mindell and Grigorieff, 2003). A semi-automated procedure in RELION (Scheres, 2012) was used to pick particles. Images were two-fold pixel-averaged to 2.84 Å per pixel. Images of individual INO80 (~113423) particles were picked after carefully sorting and cleaning. 2D and 3D classification and auto-refinement were performed using RELION. The initial model of human INO80 (Aramayo et al., 2018) was low-pass-filtered to 60 Å and was used as the starting model for the 3D classification. 3D refinement used gold-standard FSC calculations to avoid over-fitting, and reported resolutions are based on the FSC 0.143 criterion. The INO80 (~25896) ‘good’ particles group was used to polish the refinement. A soft mask was used during the auto-refinement procedure to improve the resolution, and postprocessing was carried out by ‘postprocess procedure’ in Relion using a provided B-factor –400 and a soft spherical mask (with a 14-pixel fall-off). The final resolution reported was estimated from the masking-effect-corrected FSC curve using the FSC 0.143 criterion. All the 3D structures were displayed using Chimera (Pettersen et al., 2004). The 3D cryo-EM density map of this paper has been deposited to the Electron Microscopy Data Bank under the accession number EMD-6924.

### Assembly of actin/Arp–Nuc207 complex

Nucleosomes (Nuc207) with 30 bp linker DNA at both ends (containing 207 bp DNA) were prepared as described previously (Winkler et al., 2011). The actin/Arp–Nuc207 complex was assembled by incubation ~30 μg/ml actin/Arp module fraction with a 10-fold molar excess of Nuc207 for 2 h at 4°C in the buffer (20 mM HEPES, pH 8.0, 40 mM KOAc, 5 mM MgCl_2_, 0.1% trehalose, 2 mM DTT, and 0.01% NP-40).

## Supplementary Material

Supplementary Data

## References

[mjy062C1] Aramayo R.J., Willhoft O., Ayala R., et al. (2018). Cryo-EM structures of the human INO80 chromatin-remodeling complex. Nat. Struct. Mol. Biol. 25, 37–44. 29323271 10.1038/s41594-017-0003-7 PMC5777635

[mjy062C2] Ayala R., Willhoft O., Aramayo R.J., et al. (2018). Structure and regulation of the human INO80-nucleosome complex. Nature 556, 391–395. 29643506 10.1038/s41586-018-0021-6 PMC5937682

[mjy062C3] Bartholomew B. (2014). Regulating the chromatin landscape: structural and mechanistic perspectives. Annu. Rev. Biochem. 83, 671–696. 24606138 10.1146/annurev-biochem-051810-093157 PMC4332854

[mjy062C4] Boyer L.A., and Peterson C.L. (2000). Actin-related proteins (Arps): conformational switches for chromatin-remodeling machines? Bioessays 22, 666–672. 10878579 10.1002/1521-1878(200007)22:7<666::AID-BIES9>3.0.CO;2-Y

[mjy062C5] Cai G., Imasaki T., Takagi Y., et al. (2009). Mediator structural conservation and implications for the regulation mechanism. Structure 17, 559–567. 19368889 10.1016/j.str.2009.01.016 PMC2673807

[mjy062C6] Cao T., Sun L., Jiang Y., et al. (2016). Crystal structure of a nuclear actin ternary complex. Proc. Natl Acad. Sci. USA 113, 8985–8990. 27457955 10.1073/pnas.1602818113 PMC4987789

[mjy062C7] Chen L., Cai Y., Jin J., et al. (2011). Subunit organization of the human INO80 chromatin remodeling complex: an evolutionarily conserved core complex catalyzes ATP-dependent nucleosome remodeling. J. Biol. Chem. 286, 11283–11289. 21303910 10.1074/jbc.M111.222505 PMC3064184

[mjy062C8] Clapier C.R., and Cairns B.R. (2009). The biology of chromatin remodeling complexes. Annu. Rev. Biochem. 78, 273–304. 19355820 10.1146/annurev.biochem.77.062706.153223

[mjy062C9] Eustermann S., Schall K., Kostrewa D., et al. (2018). Structural basis for ATP-dependent chromatin remodelling by the INO80 complex. Nature 556, 386–390. 29643509 10.1038/s41586-018-0029-y PMC6071913

[mjy062C10] Flaus A., Martin D.M.A., Barton G.J., et al. (2006). Identification of multiple distinct Snf2 subfamilies with conserved structural motifs. Nucleic Acids Res. 34, 2887–2905. 16738128 10.1093/nar/gkl295 PMC1474054

[mjy062C11] Frank J., Radermacher M., Penczek P., et al. (1996). SPIDER and WEB: processing and visualization of images in 3D electron microscopy and related fields. J. Struct. Biol. 116, 190–199. 8742743 10.1006/jsbi.1996.0030

[mjy062C12] Galarneau L., Nourani A., Boudreault A.A., et al. (2000). Multiple links between the NuA4 histone acetyltransferase complex and epigenetic control of transcription. Mol. Cell 5, 927–937. 10911987 10.1016/s1097-2765(00)80258-0

[mjy062C13] Gerhold C.B., and Gasser S.M. (2014). INO80 and SWR complexes: relating structure to function in chromatin remodeling. Trends Cell Biol. 24, 619–631. 25088669 10.1016/j.tcb.2014.06.004

[mjy062C14] Gerhold C.B., Winkler D.D., Lakomek K., et al. (2012). Structure of actin-related protein 8 and its contribution to nucleosome binding. Nucleic Acids Res. 40, 11036–11046. 22977180 10.1093/nar/gks842 PMC3510490

[mjy062C15] Harata M., Oma Y., Mizuno S., et al. (1999). The nuclear actin-related protein of Saccharomyces cerevisiae, Act3p/Arp4, interacts with core histones. Mol. Biol. Cell 10, 2595–2605. 10436015 10.1091/mbc.10.8.2595 PMC25491

[mjy062C16] Harata M., Oma Y., Tabuchi T., et al. (2000). Multiple actin-related proteins of Saccharomyces cerevisiae are present in the nucleus. J. Biochem. 128, 665–671. 11011149 10.1093/oxfordjournals.jbchem.a022799

[mjy062C17] Hohn M., Tang G., Goodyear G., et al. (2007). SPARX, a new environment for Cryo-EM image processing. J. Struct. Biol. 157, 47–55. 16931051 10.1016/j.jsb.2006.07.003

[mjy062C18] Jenuwein T., and Allis C.D. (2001). Translating the histone code. Science 293, 1074–1080. 11498575 10.1126/science.1063127

[mjy062C19] Jonsson Z.O., Jha S., Wohlschlegel J.A., et al. (2004). Rvb1p/Rvb2p recruit Arp5p and assemble a functional Ino80 chromatin remodeling complex. Mol. Cell 16, 465–477. 15525518 10.1016/j.molcel.2004.09.033

[mjy062C20] Kapoor P., Chen M., Winkler D.D., et al. (2013). Evidence for monomeric actin function in INO80 chromatin remodeling. Nat. Struct. Mol. Biol. 20, 426–432. 23524535 10.1038/nsmb.2529 PMC3618487

[mjy062C21] Kastner B., Fischer N., Golas M.M., et al. (2008). GraFix: sample preparation for single-particle electron cryomicroscopy. Nat. Methods 5, 53–55. 18157137 10.1038/nmeth1139

[mjy062C22] Kornberg R.D. (1974). Chromatin structure: a repeating unit of histones and DNA. Science 184, 868–871. 4825889 10.1126/science.184.4139.868

[mjy062C23] Li X., Mooney P., Zheng S., et al. (2013). Electron counting and beam-induced motion correction enable near-atomic-resolution single-particle cryo-EM. Nat. Methods 10, 584–590. 23644547 10.1038/nmeth.2472 PMC3684049

[mjy062C24] Lin C.L., Chaban Y., Rees D.M., et al. (2017). Functional characterization and architecture of recombinant yeast SWR1 histone exchange complex. Nucleic Acids Res. 45, 7249–7260. 28499038 10.1093/nar/gkx414 PMC5499540

[mjy062C25] Liu X., Li M., Xia X., et al. (2017). Mechanism of chromatin remodelling revealed by the Snf2-nucleosome structure. Nature 544, 440–445. 28424519 10.1038/nature22036

[mjy062C26] Luger K., Mader A.W., Richmond R.K., et al. (1997). Crystal structure of the nucleosome core particle at 2.8 A resolution. Nature 389, 251–260. 9305837 10.1038/38444

[mjy062C27] Mindell J.A., and Grigorieff N. (2003). Accurate determination of local defocus and specimen tilt in electron microscopy. J. Struct. Biol. 142, 334–347. 12781660 10.1016/s1047-8477(03)00069-8

[mjy062C28] Mizuguchi G., Shen X., Landry J., et al. (2004). ATP-driven exchange of histone H2AZ variant catalyzed by SWR1 chromatin remodeling complex. Science 303, 343–348. 14645854 10.1126/science.1090701

[mjy062C29] Nguyen V.Q., Ranjan A., Stengel F., et al. (2013). Molecular architecture of the ATP-dependent chromatin-remodeling complex SWR1. Cell 154, 1220–1231. 24034246 10.1016/j.cell.2013.08.018 PMC3776929

[mjy062C30] Papoulas O., Beek S.J., Moseley S.L., et al. (1998). The Drosophila trithorax group proteins BRM, ASH1 and ASH2 are subunits of distinct protein complexes. Development 125, 3955–3966. 9735357 10.1242/dev.125.20.3955

[mjy062C31] Peterson C.L., and Laniel M.A. (2004). Histones and histone modifications. Curr. Biol. 14, R546–R551. 15268870 10.1016/j.cub.2004.07.007

[mjy062C32] Pettersen E.F., Goddard T.D., Huang C.C., et al. (2004). UCSF Chimera--a visualization system for exploratory research and analysis. J. Comput. Chem. 25, 1605–1612. 15264254 10.1002/jcc.20084

[mjy062C33] Radermacher M. (1988). Three-dimensional reconstruction of single particles from random and nonrandom tilt series. J. Electron Microsc. Tech. 9, 359–394. 3058896 10.1002/jemt.1060090405

[mjy062C34] Saravanan M., Wuerges J., Bose D., et al. (2012). Interactions between the nucleosome histone core and Arp8 in the INO80 chromatin remodeling complex. Proc. Natl Acad. Sci. USA 109, 20883–20888. 23213201 10.1073/pnas.1214735109 PMC3529010

[mjy062C35] Scheres S.H. (2012). RELION: implementation of a Bayesian approach to cryo-EM structure determination. J. Struct. Biol. 180, 519–530. 23000701 10.1016/j.jsb.2012.09.006 PMC3690530

[mjy062C36] Shen X., Mizuguchi G., Hamiche A., et al. (2000). A chromatin remodelling complex involved in transcription and DNA processing. Nature 406, 541–544. 10952318 10.1038/35020123

[mjy062C37] Shen X., Ranallo R., Choi E., et al. (2003). Involvement of actin-related proteins in ATP-dependent chromatin remodeling. Mol. Cell 12, 147–155. 12887900 10.1016/s1097-2765(03)00264-8

[mjy062C38] Singleton M.R., and Wigley D.B. (2002). Modularity and specialization in superfamily 1 and 2 helicases. J. Bacteriol. 184, 1819–1826. 11889086 10.1128/JB.184.7.1819-1826.2002 PMC134918

[mjy062C39] Smith C.L., and Peterson C.L. (2005). ATP-dependent chromatin remodeling. Curr. Top. Dev. Biol. 65, 115–148. 15642381 10.1016/S0070-2153(04)65004-6

[mjy062C40] Szerlong H., Hinata K., Viswanathan R., et al. (2008). The HSA domain binds nuclear actin-related proteins to regulate chromatin-remodeling ATPases. Nat. Struct. Mol. Biol. 15, 469–476. 18408732 10.1038/nsmb.1403 PMC2810487

[mjy062C41] Tosi A., Haas C., Herzog F., et al. (2013). Structure and subunit topology of the INO80 chromatin remodeler and its nucleosome complex. Cell 154, 1207–1219. 24034245 10.1016/j.cell.2013.08.016

[mjy062C42] Voss N.R., Yoshioka C.K., Radermacher M., et al. (2009). DoG Picker and TiltPicker: software tools to facilitate particle selection in single particle electron microscopy. J. Struct. Biol. 166, 205–213. 19374019 10.1016/j.jsb.2009.01.004 PMC2768396

[mjy062C43] Watanabe S., Tan D., Lakshminarasimhan M., et al. (2015). Structural analyses of the chromatin remodelling enzymes INO80-C and SWR-C. Nat. Commun. 6, 7108. 25964121 10.1038/ncomms8108 PMC4431590

[mjy062C44] Winkler D.D., Muthurajan U.M., Hieb A.R., et al. (2011). Histone chaperone FACT coordinates nucleosome interaction through multiple synergistic binding events. J. Biol. Chem. 286, 41883–41892. 21969370 10.1074/jbc.M111.301465 PMC3308894

[mjy062C45] Xia X., Liu X., Li T., et al. (2016). Structure of chromatin remodeler Swi2/Snf2 in the resting state. Nat. Struct. Mol. Biol. 23, 722–729. 27399259 10.1038/nsmb.3259

[mjy062C46] Zhao K., Wang W., Rando O.J., et al. (1998). Rapid and phosphoinositol-dependent binding of the SWI/SNF-like BAF complex to chromatin after T lymphocyte receptor signaling. Cell 95, 625–636. 9845365 10.1016/s0092-8674(00)81633-5

