# Supplementary material for: Structure and functional interactions of INO80 actin/Arp module
Source: J Mol Cell Biol. 2018 Nov 2;11(5):345–55. doi: 10.1093/jmcb/mjy062 (PMC6548343; doi:10.1093/jmcb/mjy062)
Supplement: Supplementary Data [file mjy062_supplementary_material.pdf]

Supplementary material

Structure and functional interactions of INO80 actin/Arp module

Xuan Zhang[1], Xuejuan Wang[1,*], Zhihui Zhang[1], and Gang Cai[1, 2,*]

[1] Hefei National Laboratory for Physical Sciences at Microscale and School of Life Sciences, University of Science & Technology of China, Hefei 230026, China;

[2] CAS Center for Excellence in Molecular Cell Science, Chinese Academy of Sciences, Hefei 230026, China.

* Correspondence to: xuejuan@ustc.edu.cn (X. Wang, ORCID #: 0000-0001-6493-9971); gcai@ustc.edu.cn (G. Cai, ORCID #: 0000-0001-8622-3907)

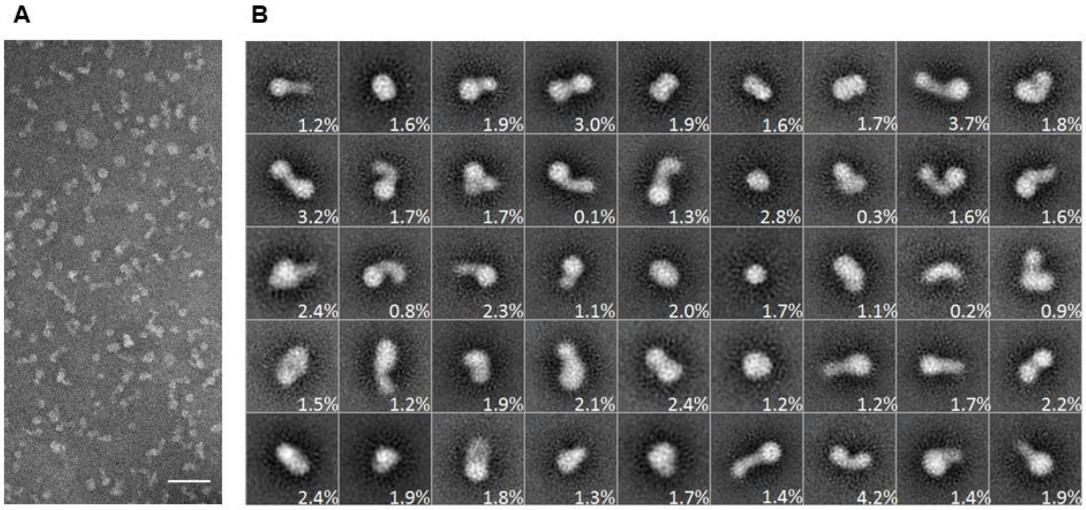

Supplementary Figure S1. Negative stain analysis of the INO80 complex. (**A**) A typical EM micrograph of INO80 complex preserved under negative stain. Scale bar, 50 nm. (**B**) Typical 2D class averages obtained after reference-free alignment and classification of images of INO80 complex. The percentages of each class are indicated below the averages.

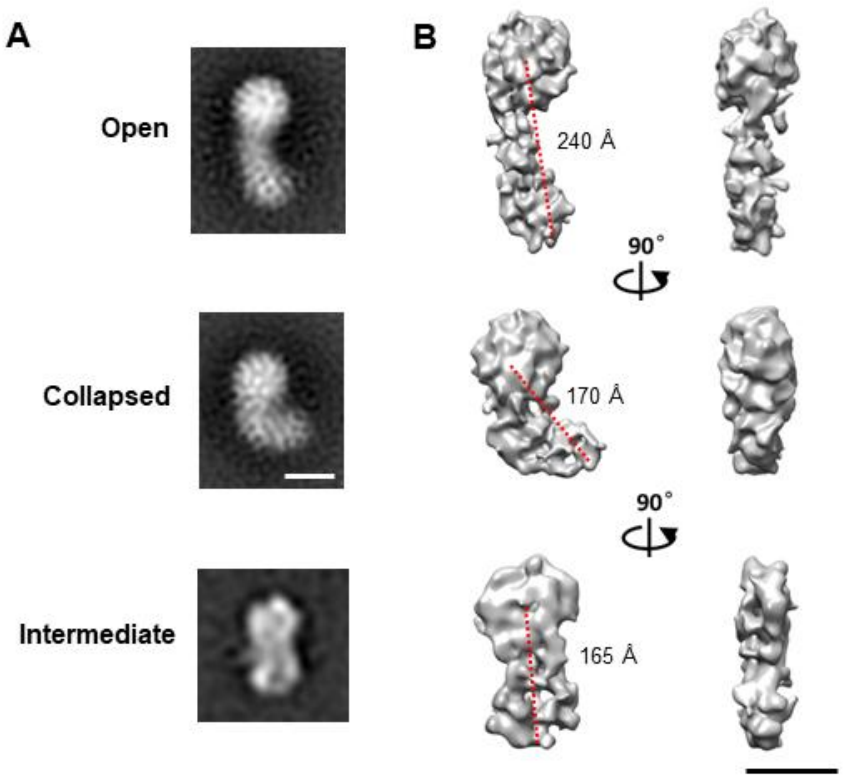

Supplementary Figure S2. Structure of the yeast INO80 complex. (**A**) Two-dimensional (2D) class averages of the INO80 complex. Three different conformations were identified through reference-free alignment and classification of EM images. Scale bar, 100 Å. (**B**) Three-dimensional (3D) reconstructions of the INO80 complex in the open, collapsed and intermediate conformations. The red dotted lines indicate the distance from center of the head region to the tip of the tail.

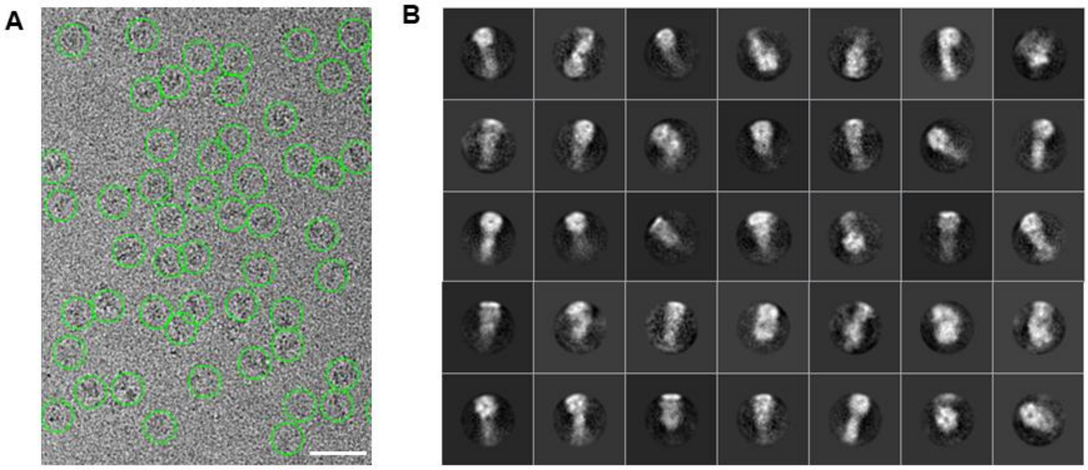

Supplementary Figure S3. Preliminary cryo-EM analysis of the INO80 complex. (**A**) A representative, motion-corrected EM micrograph of the INO80 complex preserved in vitrified ice. (**B**) Representative 2D reference free class averages of the INO80 complex.

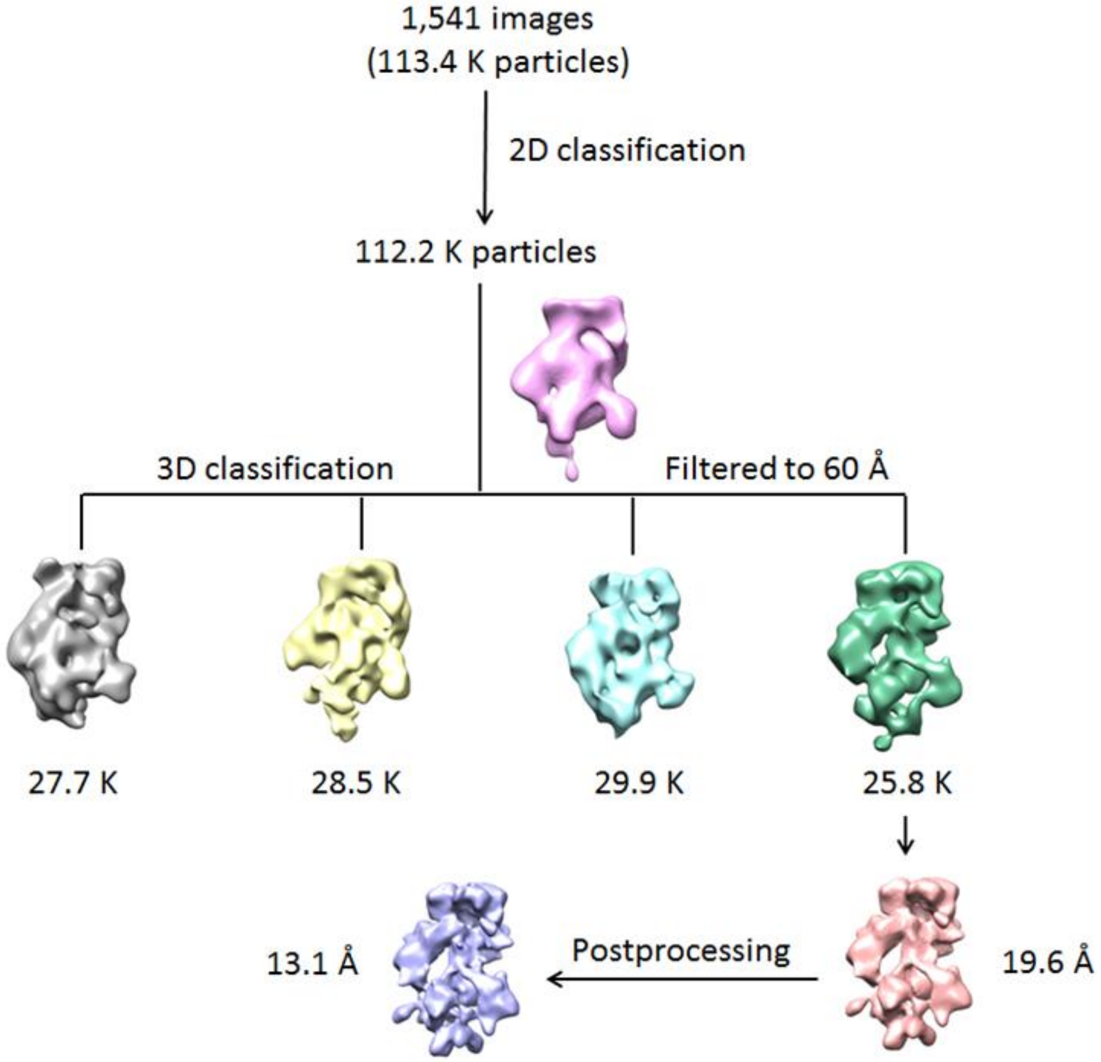

Supplementary Figure S4. Cryo-EM reconstruction process. Diagram of classification and refinement of the INO80 complex. After semi-auto picking and manual screening, a total of 113.4 K particles were subjected to several rounds of 2D classifications. The remaining 112.2 K particles were subjected to 3D classification. The published cryo-EM structure of human INO80 complex (Aramayo et al., 2018) was filtered to 60 Å and used as an initial reference for 3D classification. A final set of particles (class 4) was subjected to 3D refinement. The resolution of the final cryo-EM density map was estimated to be 13.1 Å.

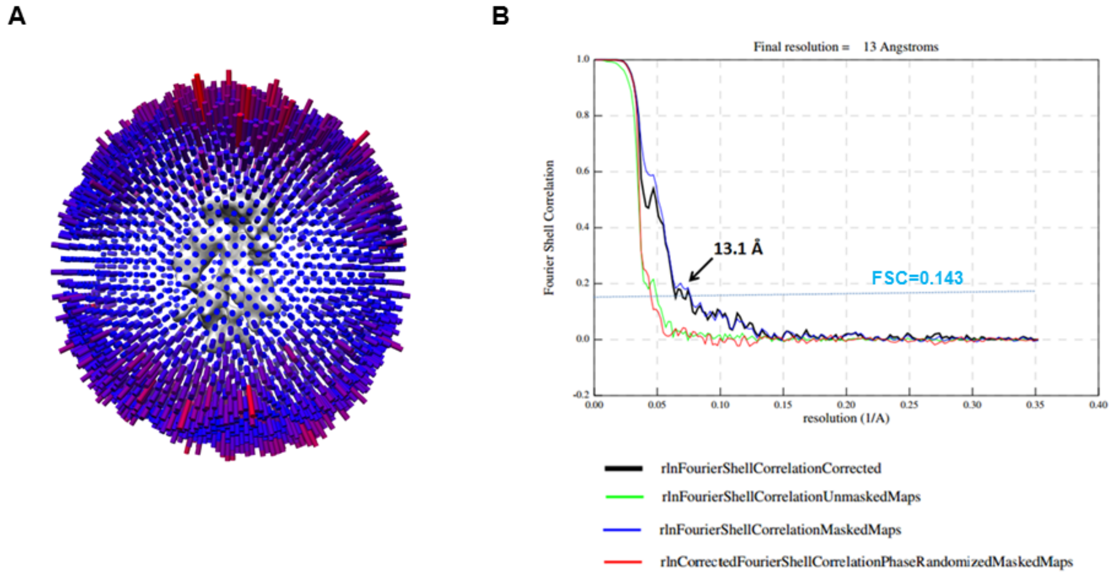

Supplementary Figure S5. The INO80 Cryo-EM reconstruction. (**A**) Angular distribution of all particles included in the final 3D reconstruction of the INO80 complex. (**B**) Gold-standard Fourier Shell Correlation (FSC) curve of the cryo-EM map revealed the final resolution of the reconstruction with the 0.143 FSC criterion was 13.1 Å.

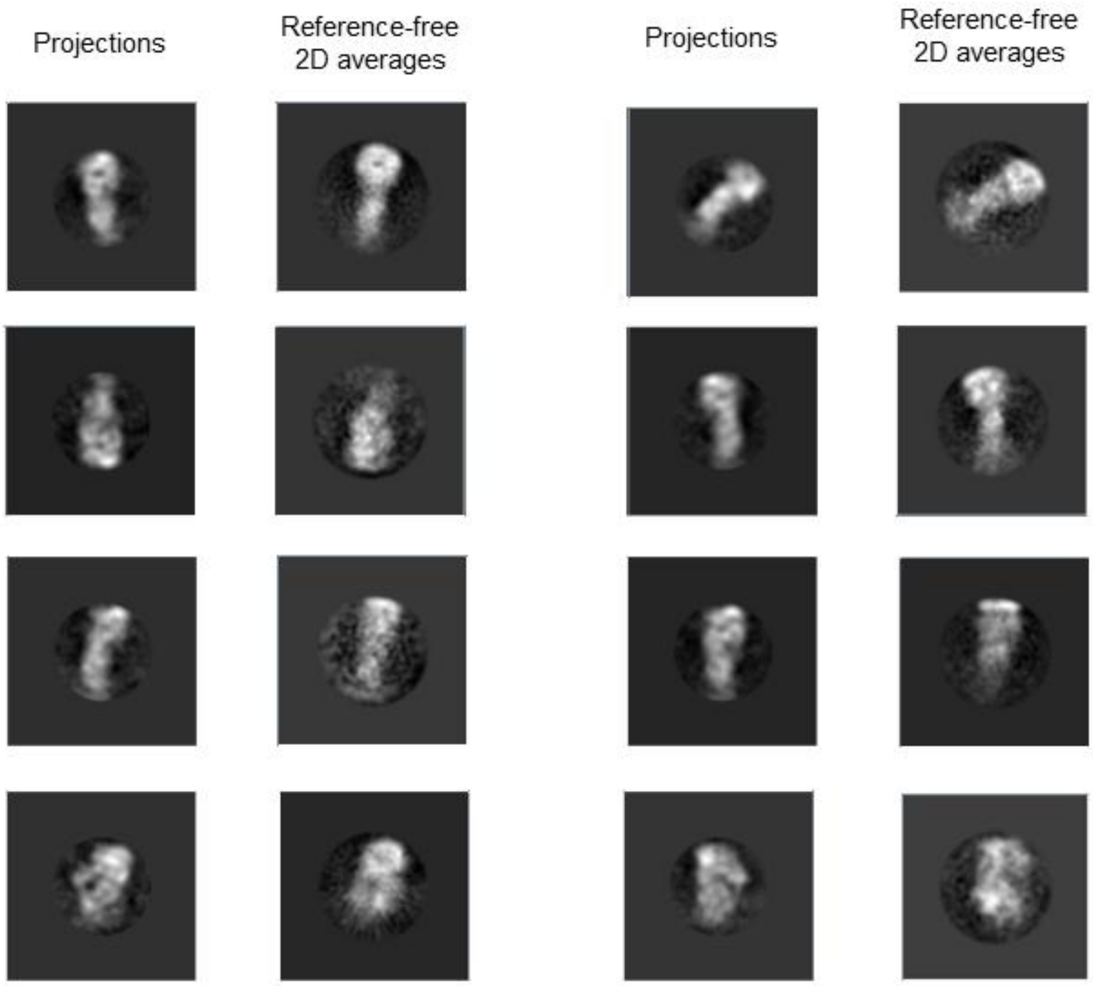

Supplementary Figure S6. Close correspondence between projections of the INO80 cryo-EM structure and 2D reference-free class averages.

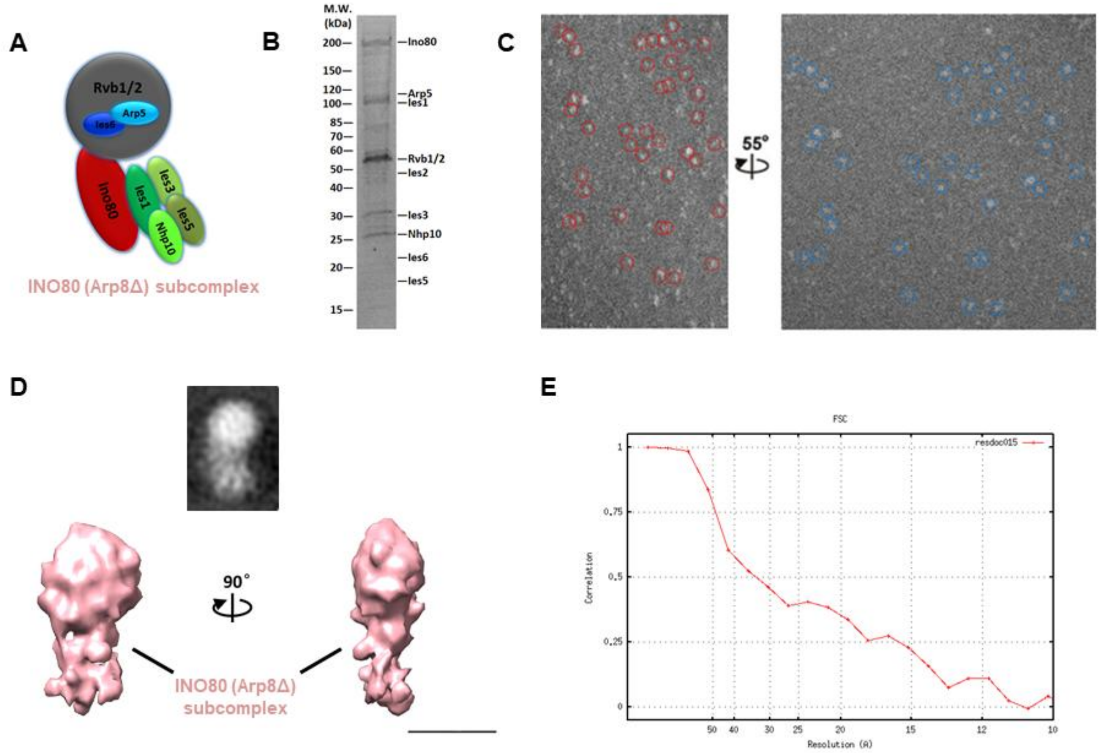

Supplementary Figure S7. 3D reconstruction of the INO80 sub-complex omitting the actin/Arp module. (**A** and **B**) Schematic view and SDS-PAGE analysis of the INO80 sub-complex omitting the actin/Arp module, which is purified from the Arp8Δ deletion mutant**.** (**C**) Tilt pair (0° and –55°) micrographs of the negatively stained INO80 sub-complex. Raw images are overlaid with circles marking the corresponding particles picked from the pair of micrographs. (**D**) Representative 2D class average and two different views of the 3D reconstruction of the INO80 sub-complex (hot pink surface). (**E**) Fourier Shell Correlation (FSC) curve for the RCT reconstruction according to the FSC = 0.5 criterion. The final resolution is estimated to be 33.2 Å.

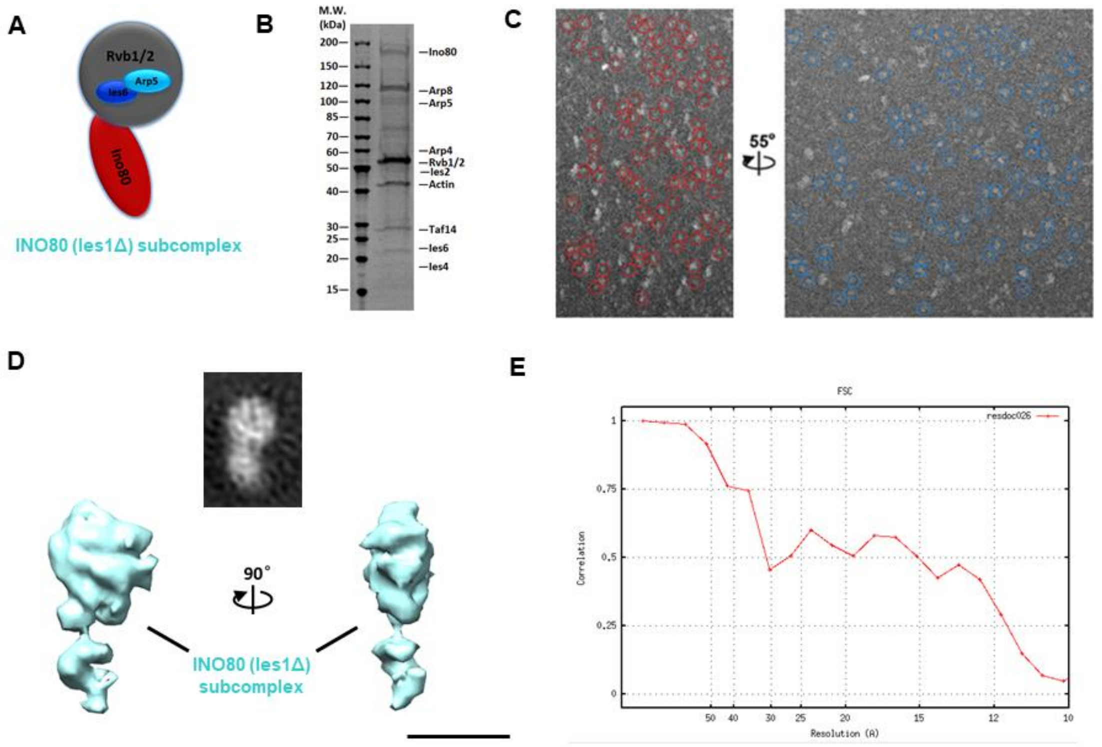

Supplementary Figure S8. 3D reconstruction of the INO80 sub-complex omitting the Nhp10 and actin/Arp modules. (**A** and **B**) Schematic view and SDS-PAGE analysis of the INO80 sub-complex purified from the Ies1Δ mutants. (**C**) Tilt pair (0° and –55°) micrographs of the negatively stained INO80 sub-complex. Raw images are overlaid with circles marking the corresponding particles picked from the pair of micrographs. (**D**) Representative 2D class average and different views of the 3D reconstruction of the INO80 sub-complex (sky blue surface) missing the Nhp10 and actin/Arp modules. (**E**) Fourier Shell Correlation (FSC) curve for the RCT reconstruction according to the FSC = 0.5 criterion. The final resolution is estimated to be 31.0 Å.

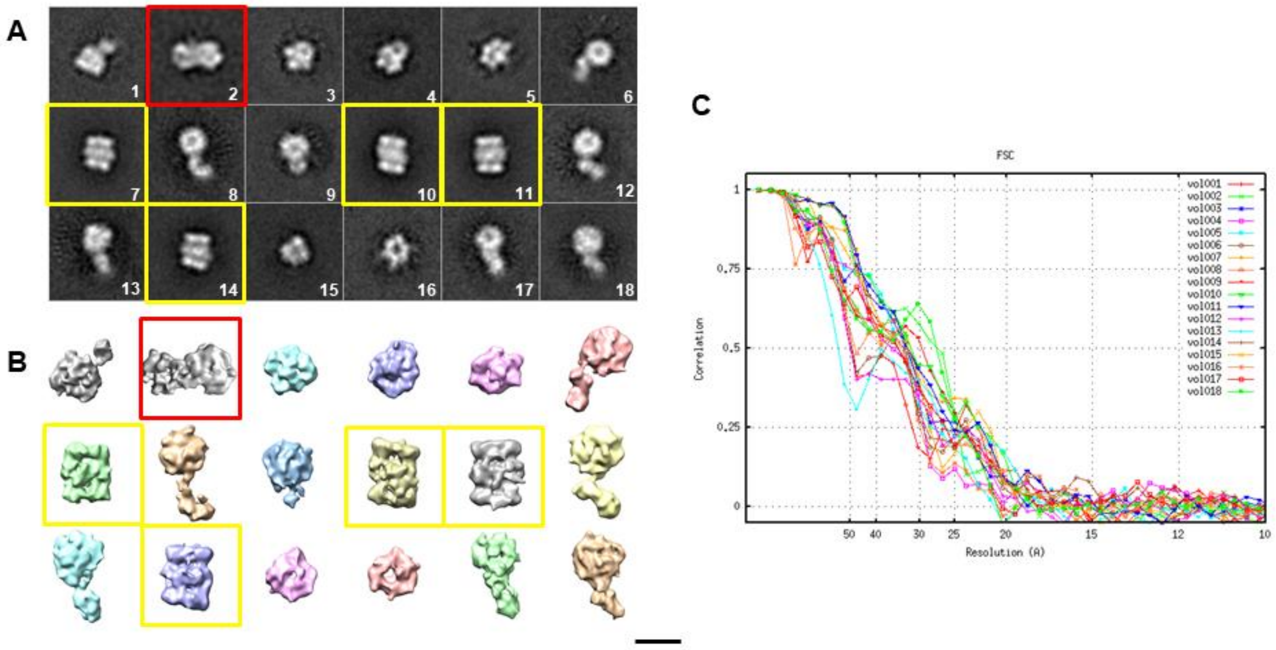

Supplementary Figure S9. EM analysis of INO80 and its sub-complexes co-purified with the INO80 complex without cross-linking. (**A**) 2D EM analysis of the INO80 and its sub-complexes. Representative 2D class averages were identified through iterative reference-free alignment and classification of the EM images. (**B**) 3D reconstructions of the INO80 and its sub-complexes showing various compositional and conformational states. The 2D class averages and 3D reconstructions of the Rvb1/Rvb2 dodecamer were identified with yellow squares. The INO80 Intermediate conformer is denoted by a red square. (**C**) Fourier Shell Correlation (FSC) curve for the RCT reconstructions according to the FSC = 0.5 criterion. The resolutions are mostly within the range of 30–40 Å.

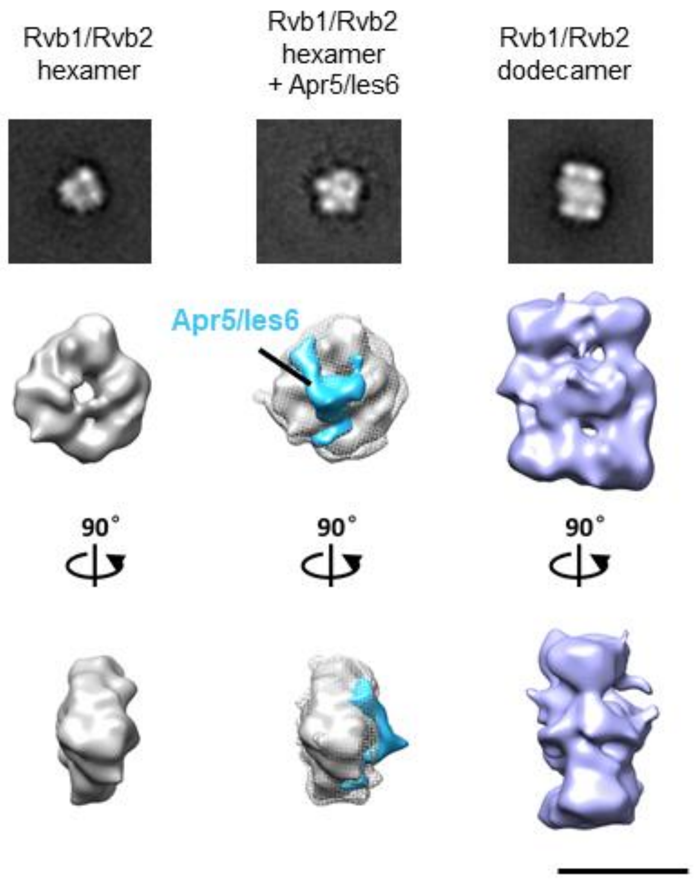

Supplementary Figure S10. 2D and 3D structures of the Rvb1/Rvb2 hexamer, Rvb1/Rvb2 hexamer + Arp5/Ies6 module and Rvb1/Rvb2 dodecamer, which are co-purified with the INO80 complex. The Arp5/Ies6 density is calculated by subtracting the density of the Rvb1/Rvb2 hexamer from the Rvb1/Rvb2 hexamer + Arp5/Ies6 volume, which is highlighted by the cyan surface.

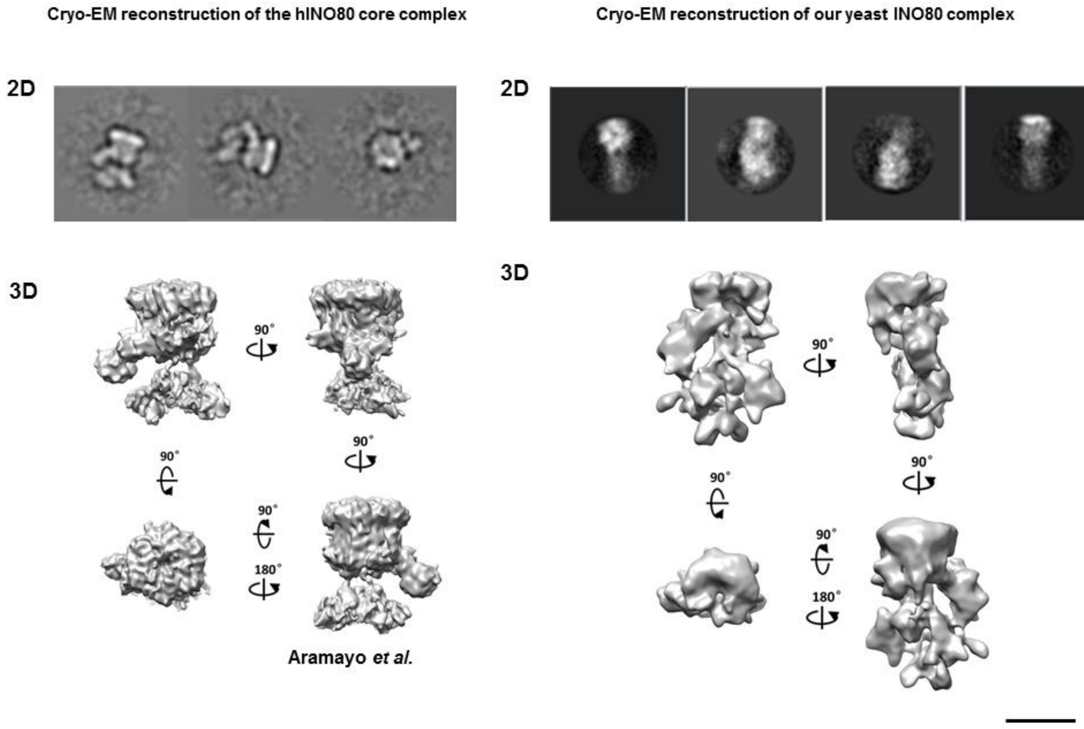

Supplementary Figure S11. Comparison of the human INO80 cryo-EM reconstruction with that of yeast INO80.

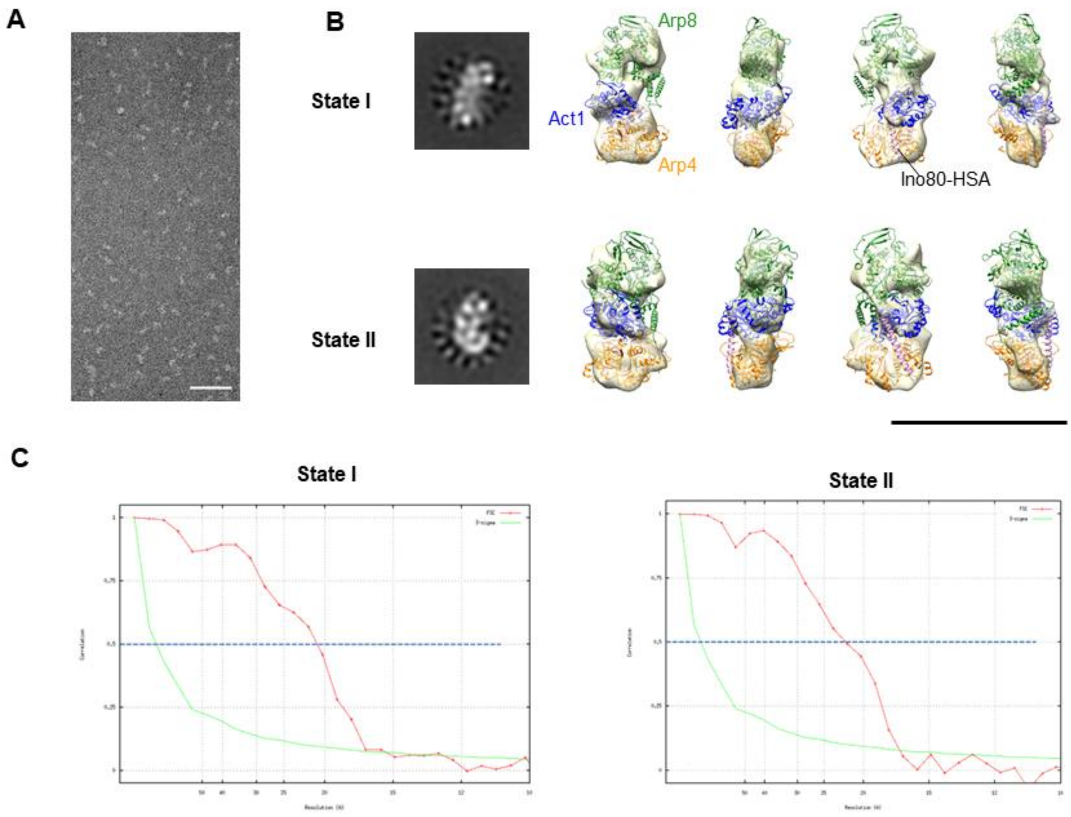

Supplementary Figure S12. EM reconstruction of the endogenously prepared actin/Arp module. (**A**) A typical EM micrograph of actin/Arp module preserved under negative stain. Scale bar, 50 nm. (**B**) 2D and 3D reconstructions of actin/Arp module in two conformational states. Different views of the 3D reconstruction rigid-body fitted the crystal structural models of the Arp8 (PDBID:4AM6) and actin-Arp4-HSA (PDBID:5I9E). This location is in agreement with crosslinks of Arp8 to actin-Arp4-HSA (Tosi et al., 2013). (**C**) Fourier Shell Correlation (FSC) curve for the RCT reconstructions according to the FSC = 0.5 criterion. The final resolution of the two conformers are estimated to be 21 Å and 23 Å, respectively.

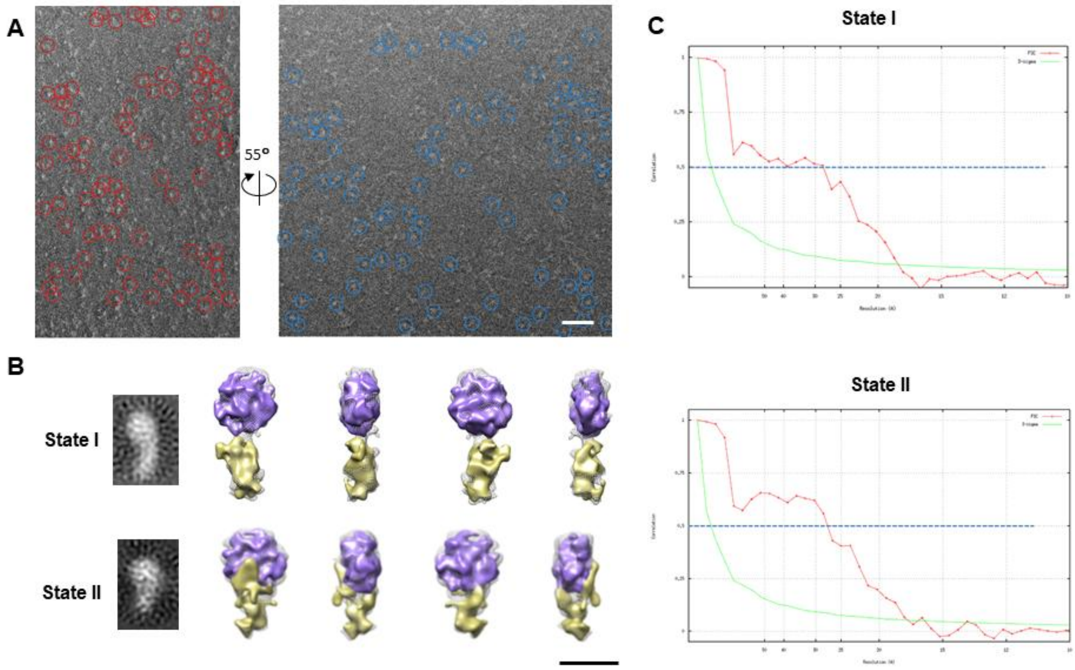

Supplementary Figure S13. EM reconstruction of the actin/Arp-Nuc207 assembly. (**A**) Tilt pair (0° and –55°) micrographs of the negatively stained actin/Arp-Nuc207 assembly. Raw images are overlaid with circles marking the corresponding particles picked from the pair of micrographs. (**B**) 2D and 3D EM reconstructions of the actin/Arp-Nuc207 assembly in two different binding states. The nucleosome structure (PDBID:2PYO) could be rigid-body fitted the EM structures and the actin/Arp densities could be identified by subtracting the nucleosome densities. Different views of the 3D reconstructions with the nucleosome and actin/Arp module densities segmented. (**C**) Fourier Shell Correlation (FSC) curve for the RCT reconstructions according to the FSC = 0.5 criterion. The final resolution of the two conformers are estimated to be 28 Å and 27 Å, respectively.
